# Ground and First Excited States of the NaSr Molecule: Experimental and Theoretical Study

**DOI:** 10.1021/acs.jpca.5c01878

**Published:** 2025-05-08

**Authors:** Jacek Szczepkowski, Marcin Gronowski, Matylda Olko, Romain Vexiau, Michał Tomza, Olivier Dulieu, Paweł Kowalczyk, Włodzimierz Jastrzebski

**Affiliations:** † 86906 Institute of Physics, Polish Academy of Sciences, al. Lotników 32/46, 02-668 Warsaw, Poland; ‡ Institute of Theoretical Physics, Faculty of Physics, 49605 University of Warsaw, ul. Pasteura 5, 02-093 Warszawa, Poland; § Laboratoire Aimé Cotton, 69255 CNRS,Université Paris-Saclay, Bât. 505, rue Aimé Cotton, F-91405 Orsay, Cedex, France; ∥ Institute of Experimental Physics, Faculty of Physics, University of Warsaw, ul. Pasteura 5, 02-093 Warszawa, Poland

## Abstract

We report the first spectroscopic investigation of the NaSr molecule. Spectra related to the B(2)^2^Σ^+^ → X(1)^2^Σ^+^ transition were observed with partial rotational resolution by thermoluminescence and laser-induced fluorescence techniques. Simultaneously, potential energy curves of the lowest electronic states of NaSr and transition dipole moments were calculated by using two different theoretical approaches. Comparison with theoretical results allowed to interpret the experimental spectra and deduce the salient molecular constants of the X(1)^2^Σ^+^ and B(2)^2^Σ^+^ states. Reliability of the employed theoretical methods was tested.

## Graphical abstract

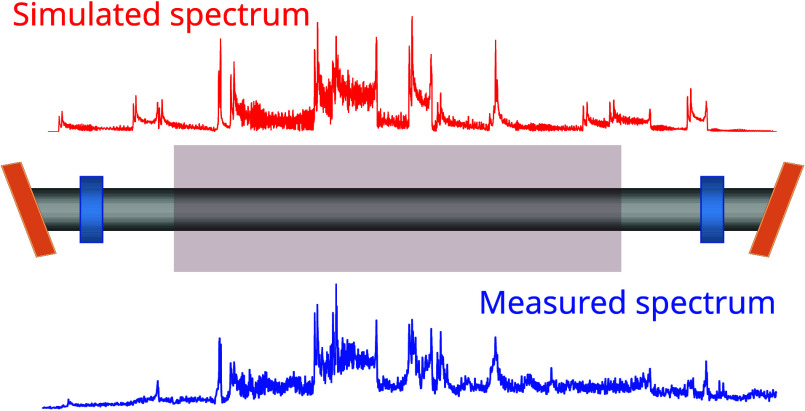

## 1 Introduction

In recent years, a growing interest toward open-shell heteronuclear molecules can be noticed. These molecules attract particular attention from physicists involved in experiments under ultracold conditions because they are known as “doubly polar molecules” having both electric and magnetic permanent dipole moments. Such properties provide a unique possibility for quantum control and simulations. Open-shell molecules, built of one alkali metal atom and one alkaline earth-like atom, have been proposed as a subject to study many-body quantum systems as dipolar quantum gases,^1^ and have been considered for measurements e.g., of parity violating nuclear anapole moments,^2^ time variation of the electron-to-proton mass ratio^3^ or electric dipole moment of electron.^4^

**Figure 1:**
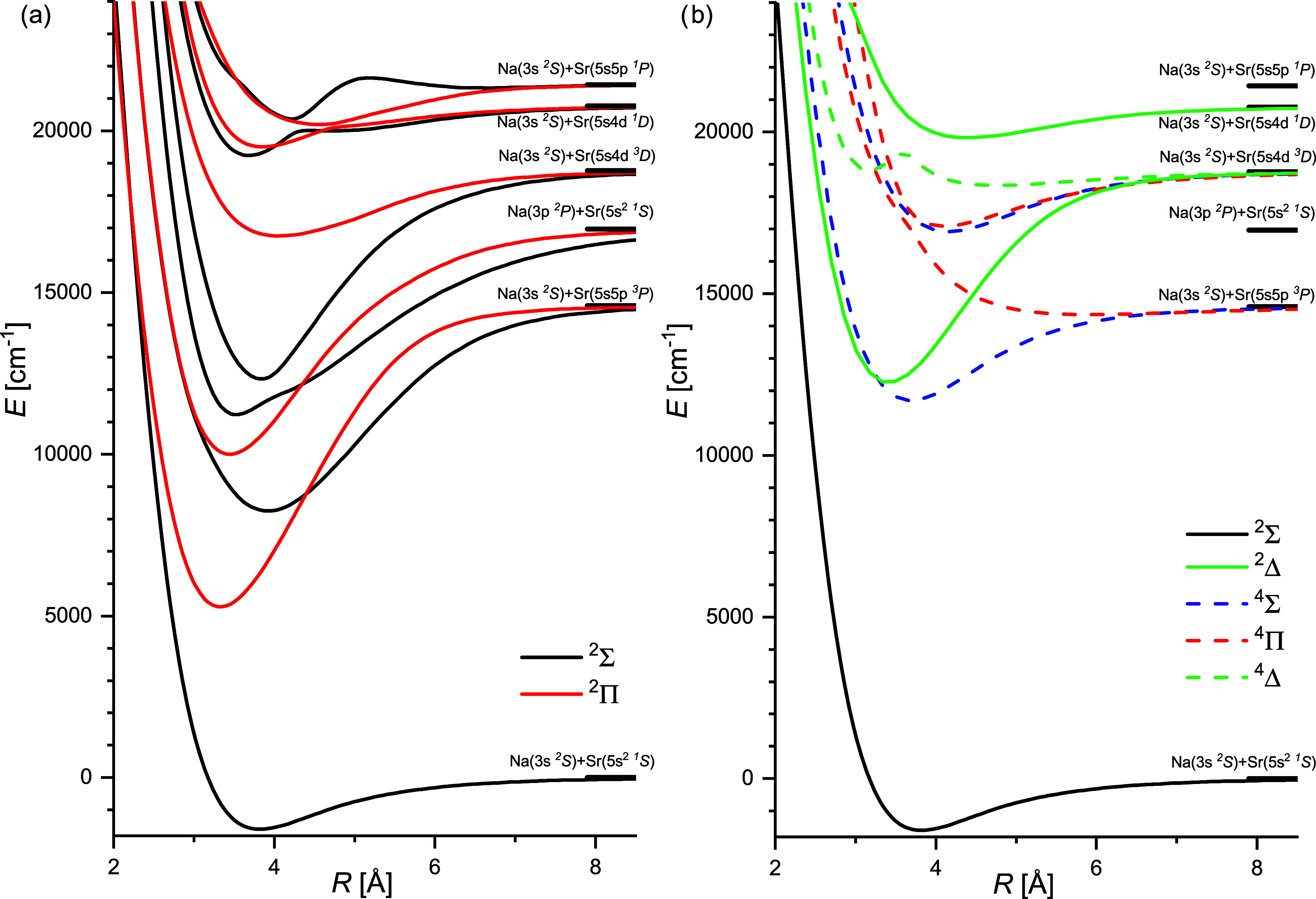
PECs of the ground state and excited states of NaSr up to the Na­(3s ^2^ *S*)+Sr­(5s5p ^1^ *P*) dissociation limit calculated with the (ECP + CPP)­FCI method: (a) for states accessible by one photon transitions from the ground state and (b) for states to which such transitions are forbidden. To keep the same scale of the panels, the ground state potential curve is depicted in both of them.

**Table 1:** Total Number of Generated Slater Determinants for Molecular Symmetries *Σ* ^+^, *Π*, and Δ, Shared in Doublet and Quartet Spin Multiplicities Resulting from the (ECP + CPP)­FCI Method

symmetry	total	doublet	quartet
Σ^+^	132,792	90,013	42,779
Π	123,684	83,182	40,502
Δ	114,840	76,923	37,917

**Table 2:** Spectroscopic Constants of the Electronic States of NaSr Obtained from Theoretical Calculations (See the Text for the Details)^a^

state	*T*_e_ [cm^–1^]	D [cm^–1^]	*R*_e_ [Å]	*B*_e_ [cm^–1^]	*D*_e_ [cm^–1^]	ω_e_ [cm^–1^]
composite CC (present paper)						
X(1)^2^Σ^+^	0	1640	3.869	0.0618	1.1	92.7
B(2)^2^Σ^+^	10227	6116	3.954	0.0592	0.8	103.1

(ECP + CPP)FCI (present paper)						
X(1)^2^Σ^+^	0	1597	3.823	0.0633	1.4	86.1
B(2)^2^Σ^+^	9951	6348	3.922	0.0601	0.8	102.8

MRCI (Pototschnig et al.^12^)						
X(1)^2^Σ^+^	0	1730	3.844	0.0627	1.3	87.6
B(2)^2^Σ^+^	9907	6526	3.988	0.0583	0.7	103.3

MRCI (Zeid et al.^13^)						
X(1)^2^Σ^+^	0	1657	3.940	0.0597	1.	85.9
B(2)^2^Σ^+^	10667	5693	4.139	0.0541	0.7	99.7

a The constants correspond to the Na^88^Sr isotopic combination. D Stands for dissociation energy. All PECs for the B(2)^2^Σ^+^ state were matched to the value of the centre of gravity of their asymptote Na­(3s ^2^S)+Sr­(5s5p ^3^ *P*) at *E* = 14702.582 cm^–1^, calculated from the experimental positions of multiplet components of the first excited strontium state.^25^ Note that for this reason, the listed *T*_e_ values differ from those given in the original papers.^12,13^

**Figure 2:**
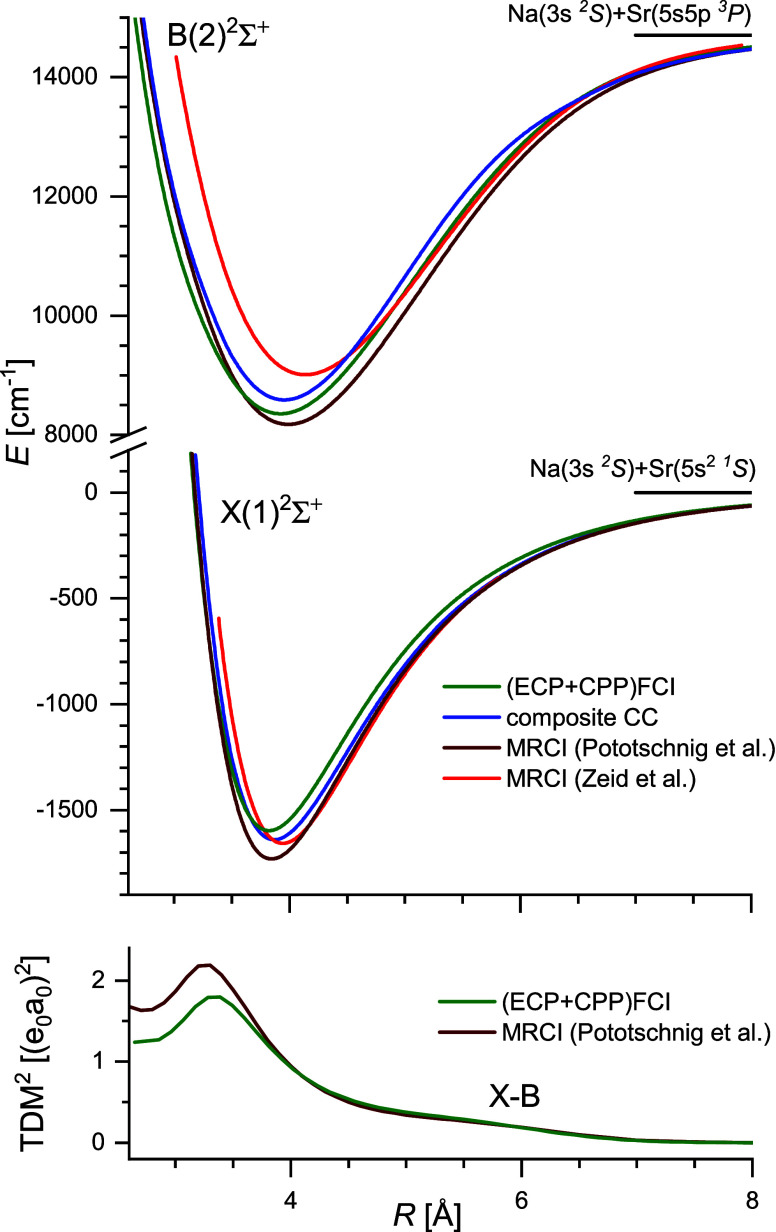
Comparison of the potential energy curves of the X(1)^2^Σ^+^ and B(2)^2^Σ^+^ states (upper panel) and TDM for transition between them (lower panel) calculated by different *ab initio* methods. Note that contrary to Table 2, zero on the energy scale is shifted to the position of the lowest atomic asymptote Na­(3s ^2^ *S*)+Sr­(5s^2 1^ *S*), to which all theoretical ground-state PECs converge. All PECs for the B(2)^2^Σ^+^ state were shifted to match the experimental position of their asymptote, Na­(3s ^2^ *S*)+Sr­(5s5p ^3^ *P*) at *E* = 14702.582 cm^–1^ (center of gravity of the multiplet components of the first excited state of strontium).^25^

**Figure 3:**
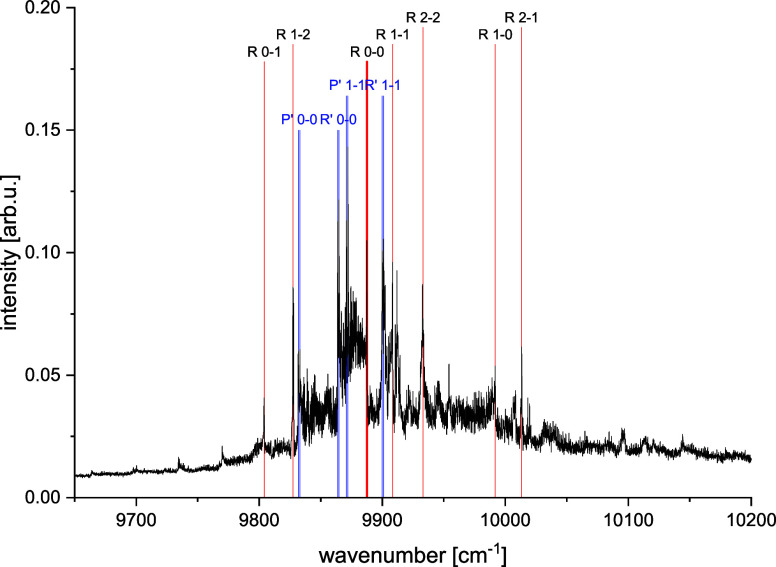
Experimental thermoluminescence spectrum of the NaSr molecule. The assigned band heads in the B → X transition are marked. For details, see Figures 4, 5 and 6

**Figure 4:**
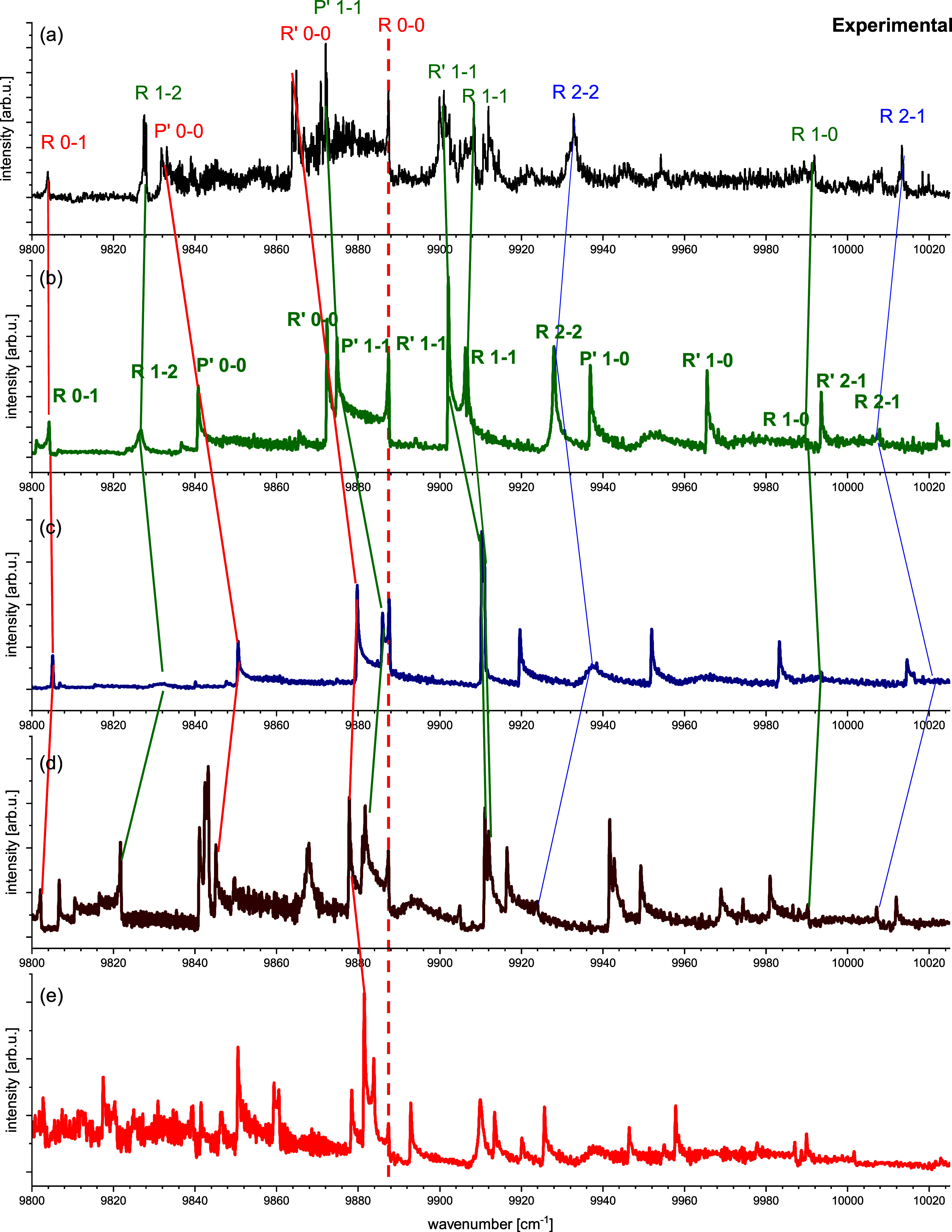
Comparison of the experimental thermoluminescence spectrum (a) with spectra obtained from simulations based on theoretical curves. The theoretical spectra were shifted so that the beginning of the 0–0 band coincides with the position of the same band in the experimental spectrum: spectra resulting (b) from the (ECP + CPP)­FCI calculations (this work) shifted by ≈26 cm^–1^, (c) from composite CC curves (this work) shifted by ≈251 cm^–1^, (d) from MRCI curves^27^ shifted by ≈71 cm^–1^, (e) from MRCI calculations^13^ shifted by ≈687 cm^–1^. The letters *R* denote positions of the band heads forming in *R* branches for transitions with *N*′ < 40, whereas the letters *P*′ and *R*′ - positions of the heads forming for transitions with *N*′ > 90 in the *P* and *R* branches, respectively. The solid lines mark the band head positions confirmed by the LIF measurements. The prominent, composite feature in panel (d) visible at about 9860 cm^–1^ corresponds to overlapping band heads *R*, *R*′, *P*′ 2–3, absent in the experimental spectrum.

**Table 3:** Deslandres Table Based on Positions of the Experimentally Observed Band Heads (in cm^–1^) in the *R* Branches for Transitions from Rotational Levels with *N*′ < 40 in the B(2)^2^ *Σ* ^+^ State of the NaSr Molecule

	*v*′ = 0		1		2
*v*″ = 0	9887.41	104.43	9991.84		
	83.59		83.49		
1	9803.82	104.53	9908.35	104.95	10013.30
			80.35		80.4
2			9828.00	104.9	9932.90

**Figure 5:**
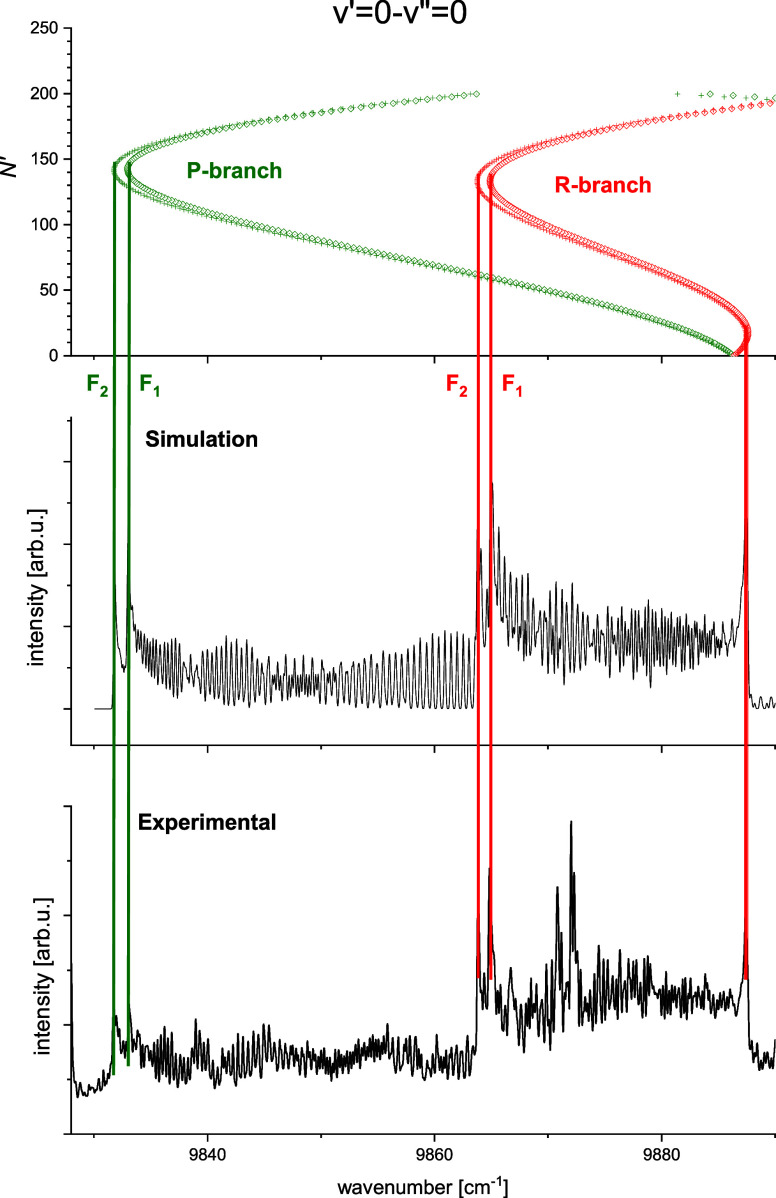
Fit of the band head positions in the 0–0 band. Rotational constants *B*_v_, *D*_v_, and *H*_v_ of the excited *v*′ = 0 level were adjusted to obtain coincidence of the heads in the experimental and simulated spectra. Constants of the *v*″ = 0 level were fixed at the values obtained from the (ECP + CPP)­FCI calculations reported in this work. The vertical lines indicate positions of the heads for the *R* branch (red lines) and the *P* branch (olive lines). The symbols *F*_1_ and *F*_2_ mark the spin-rotation components of the branches.

**Table 4:** Experimentally Determined Positions of Band Heads (in cm^–1^) Resolved into *F*_1_ and *F*_2_ Components

*v*′–*v*″	branch	*F*_1_	*F*_2_
0–0	*R*(*N*′ > 90)	9864.86	9863.85
0–0	*P*(*N*′> 90)	9833.09	9831.76
1–1	*R*(*N*′ > 90)	9901.02	9899.92
1–1	*P*(*N*′ > 90)	9872.04	9870.83
2–2	*P*(*N*′ > 90)	9911.94	9910.66

Recently, several experimental groups have attempted to produce molecules of this class at ultralow temperatures, namely LiSr,^5^ RbSr,^6^ RbYb,^7^ CsYb^8^ or LiYb.^9^ However, in the case of such experiments, both at the stage of their planning and during the process of manipulating of the molecules, information concerning molecular structure, spectroscopic constants, and shapes of potential energy curves is essential, as it would allow exact calculation of energies of molecular levels. Also, the process of production of open-shell ultracold molecules, e.g., by direct laser cooling, is a challenging task in which the possibility of success strongly depends on the properties of particular molecules. The necessary information can be provided by joint efforts of experimental and theoretical groups studying the energy structure of open-shell molecules.^6,10,11^

To the best of our knowledge, no experimental studies of the NaSr molecule have been reported until now. In the present study, we were able to detect the emission spectra of NaSr by observing thermoemission from a mixture of sodium and strontium in a high-temperature oven. It provided low-resolution spectra, in which only a few rotational band heads were recognizable. An additional laser-induced fluorescence (LIF) experiment allowed one to gain some insight into the structure of the spectra. However, a reliable interpretation of them required a comparison with theoretical predictions. Since simulations based on the previous theoretical works^12,13^ did not provide an unambiguous assignment of the observed spectral features, we undertook new calculations using two different theoretical approaches. They resulted in relatively consistent sets of potential energy curves (PECs) for several electronic states of NaSr and dispelled doubts concerning the interpretation of the experimental spectra.

In the following sections, we describe the details of the two theoretical methods employed and the results of their application. Subsequently, we present the experimental procedure, observed spectra, and their assignment. Finally, the accuracy and limitations of the theoretical models, when juxtaposed with the experimental results, are discussed.

## 2 Electronic Structure Calculations

The NaSr molecule involves 49 electrons in total, making a full *ab initio* treatment of its electronic structure tedious and somehow impractical. Precise calculations with all electrons correlated are currently not feasible due to huge computational costs, slow convergence with the basis set, and insufficient stability of standard algorithms. Instead, as achieved in our previous papers on RbSr^14,15^ and KSr,^11^ we employ two different theoretical methods based on different hypotheses for the representation of the electron cloud, in order to assess the validity of the obtained PECs, and permanent and transition dipole moments (respectively PDMs an TDMs). In short, the first approach explicitly treats the three valence electrons, while the rest of them are modeled with an analytic effective potential. In the second approach, only the 28 core electrons of Sr are replaced by an effective potential, while all electrons of Na and 10 electrons of Sr are treated explicitly.

### 2.1 (ECP + CPP) FCI Method

The first approach is the same as the one presented in several earlier papers of some of the present authors.^14,15^ It relies on the modeling of the Na^+^ and Sr^2+^ closed-shell cores by an effective core potential (ECP) completed by a core-polarization potential (CPP),^16–18^ depending on parameters reported in^14^. Thus, the problem is reduced to an effective three-electon system, allowing the resolution of the electron Schrodinger equation using a full configuration interaction (FCI). It is well-known that FCI gives access to numerous excited electronic states with a consistent precision, as it has been proved in our previous papers (see, e.g.,^15^). The Gaussian orbital basis sets spanning the configuration space of the NaSr molecule are reported in ref 14. The number of generated determinants for the molecular symmetries is given in Table 1. This yields the Hund’s case (a) PECs of the electronic ground state X(1)^2^Σ^+^, as well as excited electronic states of ^2,4^Σ^+^, ^2,4^Π, and ^2,4^Δ symmetries up to the Na­(3s ^2^ *S*)+Sr­(5s5p ^1^ *P*) dissociation limit (Figure 1), and subsequently the PDMs and TDMs involving these states as a function of the internuclear distance *R*. The corresponding data are provided in the Supporting Information (SI).

### 2.2 Composite Coupled-Cluster (CCC) Calculations

The second approach employs the coupled-cluster (CC) expansion of the electronic wave function and utilizes the additivity scheme. For convenience, we call them the composite coupled-cluster (CCC) approach. This approach requires the application of a variety of methods and Gaussian basis sets. In the vicinity of the minima, we compute the ground-state interaction potential as

1 $$V_{\mathrm{int},\mathrm{gr}}=V_{\mathrm{int},\mathrm{gr}}^{\mathrm{CCSD}\left(T\right)}+\delta V_{\mathrm{int},\mathrm{gr}}^{\mathrm{CCSDT}}+\delta V_{\mathrm{int},\mathrm{gr}}^{\mathrm{CCSDT}\left(Q\right)}$$

which is equivalent to including up to noniterative quadruple excitations in the CC expansions. We precisely define all terms in Section A of SI. This or similar approach has previously been successfully exploited for, among other things, the *ab initio* reproduction of atomization energies of light molecules,^19^ rotational constants of light closed-shell molecules^20^ and ground-state properties of alkali-metal and alkaline-earth molecules,^21^ including the scattering length for sodium–lithium collision.^22^ However, its application to the electronically excited states of open-shell heavy molecules requires modification to accommodate the unavailability or poor numerical stability of the implementation of some methods. Thus, we obtain the excited-state potential energy curves in a slightly different way, as

2 $$V_{\mathrm{int},\mathrm{exc}}=V_{\mathrm{int},\mathrm{gr}}+\Delta V_{\mathrm{int}}$$

where Δ*V*_int_ describes the excitation energy, i.e., difference between the ground and excited state potential energy curves (see Section A of SI for precise definition). Our description of excited states is equivalent to including all terms up to triple excitations in the CC expansions.

We model the 1s^2^2s^2^2p^6^3s^2^3p^6^3d^10^ core of strontium by the small-core relativistic energy-consistent ECP, namely ECP28MDF,^23,24^ which is based on the atomic multiconfiguration Dirac-Coulomb-Hartree–Fock calculations. We estimate (see Section C of SI for details) that usage of such a small-core ECP may change the ground-state interaction energy of the NaSr by a few cm^–1^ and the electronic excitation energy by a few tens cm^–1^.

We apply the Duning-type basis set of increasing size from triple- to six-zeta to systematically check the energy’s convergence. In some calculations, we speed up the convergence by applying the midbond functions. In order to reduce the basis set superposition error, each interaction energy was calculated as a difference between dimer and monomer energies using a dimer basis set.

We estimate that uncertainties of *V*_int,gr_ and Δ*V*_int_ are, respectively, 40^1^ and 73 cm^–1^ in the vicinity of the minima (see Section D of SI for more details). Additional changes to potential may be introduced by spin–orbit coupling, which was neglected in all present calculations.

A comparison of the results of different theoretical approaches to the X and B states of NaSr is presented in Table 2 and Figure 2.

## 3 Experimental Section

The spectra of the NaSr molecule were recorded in two independent thermoluminescence and LIF experiments. A detailed description of the thermoluminescence experiment was already published in our previous paper,^6^ thus only a brief summary is provided here. NaSr molecules were produced in a dual-temperature heat-pipe oven, filled with 10 g of strontium (natural isotopic composition) and 10 g of sodium. The central part of the heat-pipe, containing strontium, was heated to the temperature *T* = 1100 K, and the outer parts, in which sodium was placed, were maintained at *T* = 820 K. Helium at a pressure of 30 Torr served as a buffer gas. Due to the mixing of atomic vapors in the central, hottest part of the heat-pipe, NaSr molecules were produced there. At high temperature, a thermal population of excited electronic states of NaSr was achieved. It was followed by the spontaneous emission of photons in transitions to the ground electronic state. A Bruker Vertex V80 Fourier Transform Spectrometer of spectral resolution 0.16 cm^–1^ was used to record the resulting thermoluminescence spectrum. Unresolved band heads corresponding to transitions between vibrational levels of the excited B(2)^2^Σ^+^ and the ground X(1)^2^Σ^+^ electronic states of the NaSr molecule could be recognized in the spectrum (Figure 3).

In the second experiment, LIF spectra were recorded for NaSr., This technique was already used in case of the RbSr molecule and described in ref 6. In the LIF experiment a homemade external-cavity diode laser (INNOLUME GCB1030150TC200 M gain chip) with 100 mW output power and <0.01 cm^–1^ line width was employed to populate the excited electronic B(2)^2^Σ^+^ state of NaSr molecule. Its wavelength was set subsequently to positions of chosen band heads corresponding to transitions between the X(1)^2^Σ^+^ and B(2)^2^Σ^+^ states observed during the thermoluminescence experiment (with the laser wavelength actively stabilized using the HighFinesse WS7 wavemeter). The resulting fluorescence was recorded with the same Fourier transform spectrometer. In this experiment, the temperature of the central part of the heat-pipe, containing NaSr molecules, was reduced to 900 K to increase the ratio between the LIF and the thermoluminescence signal.

## 4 Simulations of the Recorded Spectra

In the spectral range from 9650 to 10200 cm^–1^, a number of distinct band heads were observed in the thermoemission spectrum. According to theoretical predictions^12,13^ in this region the A(1)^2^Π → X(1)^2^Σ^+^ and B(2)^2^Σ^+^ → X(1)^2^Σ^+^ band systems should be observed in NaSr., In order to identify the states and vibrational levels involved in the transitions, spectrum simulations were performed based on four sets of theoretical potential energy curves available, including two sets obtained in this work. As the simulation method was described previously,^11^ we only outline here the assumptions made. All allowed transitions between the excited and ground state levels were included in the simulation. During the calculation of line intensities, the Boltzmann population distribution in the excited states, Franck–Condon factors, and Hönl-London coefficients were taken into account, as well as dipole transition moments when available. At this stage of the analysis the effect of energy level splitting due to spin-rotation interaction, which is observed for alkali metal–strontium molecules,^26^ was not taken into account, because of the lack of relevant theoretical data. Also, spin–orbit interaction is not included: it would probably lead to a global shift of the excited state PEC, as the explored spectral range is located below the crossing with the 1^2^Π state. The line shapes were described by Gaussian profiles with a half-width of 0.16 cm^–1^ corresponding to the limited resolution of the Fourier spectrometer used in the experiment. Calculations were performed for the dominant isotopologue Na^88^Sr (abundance of 82.6%). In the observed spectral region, simulations revealed only transitions between the excited B(2)^2^Σ^+^ state and the ground state. Comparison of the experimental and simulated spectra allowed a preliminary determination of the position of the heads in the two bands 0–0 and 1–1 (hereafter we adopt the *v*′ – *v*″ notation for vibrational bands). It was observed that in both bands, the heads form in the *R* branch as well as in the *P* branch. For the sake of readability, in Figure 4 only a part of the experimental spectrum corresponding to the region around the 0–0 and 1–1 bands is compared with the simulations. A comparative diagram for the entire recorded spectral region is provided in the Supporting Information.

To match the experimental and theoretical positions of the onset of the 0–0 band (Figure 4), each modeled spectrum had to be shifted on the wavenumber axis. Although the magnitude of the shift varies between methods, it does not correlate with the ability of a particular method to reproduce the overall shape of the spectrum. Only the CCC and (ECP + CPP)­FCI approaches successfully reproduce most of the spectral features. However, the required shift for the CCC method is roughly 1 order of magnitude larger than that for (ECP + CPP)­FCI. The large shift in this case arises primarily from the absence of spin–orbit coupling in the long-range region of the 2^2^Σ^+^ potential. In our *ab initio* calculations, the CCC potential curve has been aligned with the term energy of the atomic asymptote, i.e., the center of gravity of its multiplet components. Preliminary calculations that include spin–orbit effects suggest that the 2^2^Σ_1/2_^+^ state correlates with the ^3^P_1_ state of Sr, which lies approximately 198 cm^–1^ lower. Thus, nearly 80% of the energy shift required in the CCC method can probably be attributed to spin–orbit interactions in the long-range region. Conversely, assuming a simple LS coupling scheme, the unperturbed term (which corresponds to our computations) is expected to lie significantly higher in energy. This highlights that neglecting spin–orbit coupling has a substantial effect on the positions of the potential energy curves. Fortunately, the spin–orbit interaction is small near the potential minimum and therefore has little influence on the simulated spectral shapes. Surprisingly small energy shifts of the two (ECP + CPP)­FCI potential curves required to align the simulated and experimental spectra can be explained as follows. The (ECP + CPP)­FCI calculations treat all states of a given symmetry consistently, which likely leads to a partial cancellation of intrinsic errors in the electronic structure calculations when evaluating transition energies (i.e., energy differences). This compensation is not present in the CCC approach.

## 5 Analysis of the Data

The analysis of the experimental spectrum involved several steps. After identifying heads in the 0 – 0 and 1 – 1 bands based on theoretical predictions, the LIF technique was used to assign further heads. For this purpose, the laser wavelength was tuned to the position of each of the two band heads mentioned above, and the arising fluorescence spectrum was recorded using the Fourier spectrometer. Thus, excitation of the *v*′ = 0 level near the 0–0 head resulted not only in fluorescence back to *v*″ = 0 but also to *v*″ = 1, producing a band head shifted by about 80 cm^–1^ to lower energies. Excitation of the *v*′ = 1 level around the 1 – 1 head was followed by fluorescence to *v*″ = 1, but also to *v*″ = 0 and 2, the latter two recognized in the spectra as band heads shifted by about +80 and −80 cm^–1^, respectively. Finally, the position of 2–1 and 2–2 heads was determined on the basis of their nearly equal, approximately 100 cm^–1^ shifts from the corresponding 1–1 and 1–2 band heads (cf. Table 2 for theoretically predicted vibrational constants of the X(1)^2^Σ^+^ and B(2)^2^Σ^+^ states). Note that due to similarity of equilibrium distances for potentials of the X(1)^2^Σ^+^ and B(2)^2^Σ^+^ states, only transitions with Δ*v* = 0, ± 1 could be observed (so-called diagonal groups^28^). In Table 3 the identified band heads are arranged as a Deslandres table.

However, the above analysis has only been able to explain part of the features visible in the recorded spectra. The presence of other maxima, placed less regularly, suggested that in the observed bands, reversal of shading occurred, producing more than one head in each band. To verify this hypothesis, more complex analysis taking into account the rotational structure of the bands was needed. For this purpose the PGopher program^29^ was employed. Initially, the rotational structure of the 0–0 and 1–1 bands was simulated with the use of rotational constants derived from theoretical calculations. The energies of rotational levels in both states were represented as

3 $$E\left[v,N\right]=E_{v}+B_{v}N\left(N+1\right)-D_{v}{\left(N\left(N+1\right)\right)}^{2}+H_{v}{\left(N\left(N+1\right)\right)}^{4}\pm \gamma_{v}\left(N+1/2\mp 1/2\right)$$

where *E*_v_ denotes the rotational energy of a given vibrational level, rotational constants *B*_v_, *D*_v_, *H*_v_ have their usual meaning^28^ and γ_v_ is the spin-rotation coupling constant. Upper and lower signs in eq 3 refer to the *F*_1_ and *F*_2_ levels, respectively. As the shape of a rotational band depends actually on a difference of the ground and excited state constants, we fixed the X(1)^2^Σ^+^ state rotational constants on theoretical values and optimized only the constants related to the upper state. Note that the *H*_v_ constant for the B state was added as a fitting parameter to improve the quality of the fits, but we do not expect it to have any physical meaning. Having several theoretical calculations using different methods to our disposal, we performed separate optimizations for each set of the excited state constants (reported in Table 5), although the results were qualitatively the same. Details of the optimization procedure are described at length in our previous work.^11^ In the absence of theoretical prediction of the spin-rotation constant in the ground state, the value of γ in Table 5 is actually a difference of the relevant constants in the B(2)^2^Σ^+^ and X(1)^2^Σ^+^ states.

The simulations of individual bands revealed that a reversal of shading and formation of several heads is indeed occurring. As an example in the 0–0 band presented in Figure 5, we observe a red shaded head in the *R* branch formed for transitions from levels with *N*′ < 40 and two blue shaded heads in the *P* and *R* branches for transitions originating from levels with *N*′ > 90. Red shading of heads corresponding to low *N*′ values shows that the equilibrium distance *R*_e_ for the B(2)^2^Σ^+^ state is larger than that for the ground state. In the case of heads formed for transitions from high rotational levels (*N*′ > 90) splitting to spin-rotation components *F*_1_ and *F*_2_ can be observed whereas for low *N*′ only a broadening of band heads is seen. Summing up, spectral simulations of the rotational structure in the thermoluminescence spectrum allowed to identify 7 red shaded band heads formed in the *R*(*N*′ < 40) branches (Table 3) and 5 blue shaded heads in the *P*(*N*′ > 90) and *R*(*N*′ > 90) branches, each doubled by the spin-rotation interaction (Table 4).

For the purpose of easier comparison with the present and future theoretical results, the constants of Table 5 were converted to term energies *T*_e_, vibrational constants ω_e_ as well as rotational constants *B*_e_ and equilibrium distances *R*_e_ of both electronic states, listed in Table 6. As the values of molecular constants determined in the optimization process are highly correlated with the theoretical constants of the ground state, estimation of the uncertainty of the obtained values is difficult because of a lack of information about the uncertainties of the latter ones. Therefore, to determine the quality of the simulation, we derived the root-mean-square deviation of the positions of the band heads in the simulated spectrum with respect to their position in the experimental spectrum (Table 6). A comparison of the experimental spectrum with the simulated spectrum based on optimized molecular constants (ECP + CPP)­FCI (see Table 5) is presented in Figure 6.

**Figure 6:**
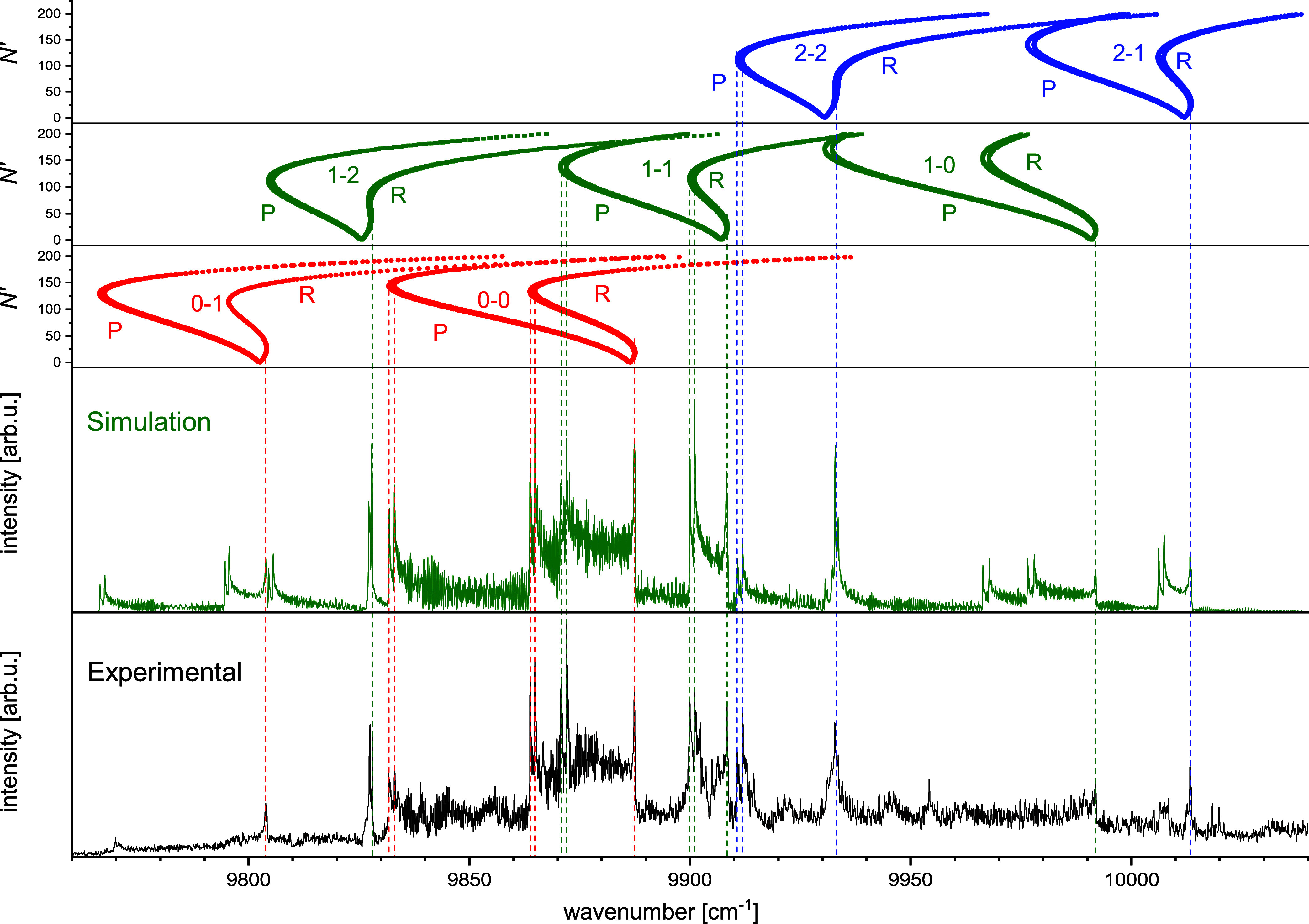
Comparison of the experimental spectrum (lower, black) with the one simulated using the PGopher program (upper, olive) based on optimized molecular constants (ECP + CPP)­FCI (see Table 5). The uppermost part of the figure illustrates the formation of multiple band heads in the spectrum. Vertical dashed lines indicate band heads identified during the spectrum analysis.

**Table 5:** Fitted Band Constants Related to Vibrational Levels of the X(1)^2^ *Σ* ^+^ and B(2)^2^ *Σ* ^+^ States of NaSr Observed in the Present Experiment Were Obtained with Rotational Constants of the Ground State Fixed at Values Resulting from Various Theoretical Methods

method	*v*	*T*_v_ [cm^–1^]	*B*_v_ [cm^–1^]	*D*_v_·10^7^ [cm^–1^]	*H*_v_·10^13^ [cm^–1^]	γ_v_·10^3^ [cm^–1^]
X(1)^2^Σ^+^ (only *T*_v_ fitted)						
(ECP + CPP)FCI^a^	0	42.8455	0.06289	1.3724	–4.6234	–
	1	126.72	0.06206	1.4182	–5.632	–
	2	208.187	0.06119	1.4668	–6.7982	–
composite CC^a^	0	44.8218	0.06137	1.2164	–6.6798	–
	1	128.865	0.06055	1.2414	–8.05	–
	2	211.082	0.05969	1.2542	–9.8981	–
MRCI^b^	0	44.8667	0.06221	1.277	–3.8005	–
	1	128.875	0.06142	1.298	–5.1897	–
	2	211.078	0.06057	1.3113	–6.6204	–
MRCI^c^	0	43.5198	0.0592	1.1228	–3.2849	–
	1	127.276	0.05848	1.1616	–4.0838	–
	2	210.327	0.05773	1.1955	–5.1994	–
B(2)^2^Σ^+^ (all parameters fitted)						
(ECP + CPP)FCI^a^	0	9929.17	0.05969	1.0404	11.731	8.304
	1	10033.7	0.05945	0.5284	–9.6444	9.318
	2	10138.6	0.05967	0.6145	–9.7338	10.65
cmposite CC^a^	0	9931.01	0.05867	1.1575	11.039	7.835
	1	10035.8	0.05825	0.66577	–4.3869	8.777
	2	10140.8	0.05879	1.036	1.0467	10.32
MRCI^b^	0	9931.03	0.05948	1.2763	16.907	7.831
	1	10035.7	0.05915	0.82135	3.6039	8.818
	2	10140.8	0.05962	0.99492	1.002	10.31
MRCI^c^	0	9929.91	0.05619	0.86056	6.0084	5.151
	1	10033.9	0.05683	1.2042	8.6614	12.39
	2	10139.2	0.05758	1.3086	0.71203	7.793

a This work.

b Ref 12.

c Ref 13.

**Table 6:** Salient Spectroscopic Constants of the X(1)^2^ *Σ* ^+^ and B(2)^2^ *Σ* ^+^ States of Na^88^Sr Obtained from the Experimental Data^a^

	X(1)^2^Σ^+^		B(2)^2^Σ^+^	
constant	theory	experimental	theory	experimental
*T*_e_ [cm^–1^]	0	0	10227^c^	9879
	0	0	9951^d^	9877
	0	0	9907^e^	9879
	0	0	10667^f^	9878
ω_e_ [cm^–1^]	92.7^c^	86.0	103.1^c^	104.2
	86.1^d^	86.4	102.8^d^	104.0
	85.9^e^	86.0	103.2^e^	104.1
	89.9^f^	84.55	97.8^f^	102.9
*B*_e_ [cm^–1^]	0.0618^c^	0.0618^b^	0.0592^c^	0.0591
	0.0633^d^	0.0633^b^	0.0601^d^	0.0599
	0.0627^e^	0.0627^b^	0.0583^e^	0.0598
	0.0597^f^	0.0597^b^	0.0541^f^	0.0560
*R*_e_ [Å]	3.869^c^	3.869^b^	3.954^c^	3.956
	3.823^d^	3.823^b^	3.922^d^	3.9300
	3.844^e^	3.844^b^	3.988^e^	3.933
	3.940^f^	3.940^b^	4.139^f^	4.064
σ_rms_ [cm^–1^]	0.47^c^			
	0.03^d^			
	0.15^e^			
	12.52^f^			

a The *σ*_rms_ reflects deviations of positions of the band heads in the simulated spectrum from positions of band heads in the experimental spectrum (see the text for details).

b Fixed to theoretical value.

c Composite CC (this work).

d (ECP + CPP)­FCI (this work).

e MRCI^12^.

f MRCI^13^.

## 6 Conclusions

In this paper, we achieved the first spectroscopic characterization of the open-shell, doubly polar molecule NaSr, using two different approaches: thermoluminescence and LIF, for transitions around 10,000 cm^–1^. We identified resonant transitions between the electronic ground state X(1)^2^Σ^+^ and the second electronic excited state B(2)^2^Σ^+^, involving the two lowest vibrational levels of each state, thus determining the minimal excitation energy of NaSr., This assignment was made possible on the basis of elaborate electronic structure calculations of two kinds, mostly differing in the method for treating the correlation between valence electrons and core electrons. Despite this difference, the two approaches yield results that are in good agreement with each other. One of the methods further provides an accurate description of the electronically excited states up to the sixth dissociation limit of NaSr, where the strontium atom is excited to the ^1^P_1_ state. We note that in spite of the difference in shift required for the simulated spectra, the shapes of the PECs are remarkably close. Actually, this is the reason we performed calculations with two methods in order to assess their validity by reciprocal comparison. While the simulation of the recorded spectrum seems to discriminate the FCI approach against the CCC one, that may well be coincidental, as the spin–orbit interaction is not taken into account in the simulation. This is the important missing ingredient of both theoretical approaches and must be included in future studies.

## Supplementary Material





## Notes

Provides a detailed description of the CCC calculations (Sections A–D), additional figures (Section E), and numerical results of both theoretical approaches (Sections F and G) (PDF)
New basis set for Sr atoms (ZIP)
