# Supplementary material for: Ground and First Excited States of the NaSr Molecule: Experimental and Theoretical Study
Source: J Phys Chem A. 2025 May 8;129(20):4496–504. doi: 10.1021/acs.jpca.5c01878 (PMC12105035; doi:10.1021/acs.jpca.5c01878)
Supplement: Supplementary file 1 [file jp5c01878_si_001.pdf]

Supporting Informaton for Publication "Ground and First Excited States of the NaSr Molecule: Experimental and Theoretical Study"

Jacek Szczepkowski,*,† Marcin Gronowski,*,‡ Matylda Olko,† Romain Vexiau,¶ Michał Tomza,‡ Olivier Dulieu,*,¶ Paweł Kowalczyk,§ and Włodzimierz Jastrzebski†

†*Institute of Physics, Polish Academy of Sciences, al. Lotników 32/46, 02-668 Warsaw, Poland*

‡*Institute of Theoretical Physics, Faculty of Physics, University of Warsaw, ul. Pasteura 5, 02-093 Warszawa, Poland*

¶*Laboratoire Aimé Cotton, CNRS,Université Paris-Saclay, Bât. 505, rue Aimé Cotton, F-91405 Orsay Cedex, France*

§*Institute of Experimental Physics, Faculty of Physics, University of Warsaw, ul. Pasteura 5, 02-093 Warszawa, Poland*

E-mail: jszczep@ifpan.edu.pl; marcin.gronowski@fuw.edu.pl; olivier.dulieu@u-psud.fr

A Composite coupled-cluster (CCC) calculations

Composite coupled-cluster (CCC) approach requires the application of a variety of methods and Gaussian basis sets. Let us denote the interaction energy for the electronic state x

obtained by the electronic structure method y using the basis set z as $V^{y/z}_{\mathrm{int},x}$. The x could be either "gr" or "exc" for the ground $X(1)^2\Sigma^+$ and excited $B(2)^2\Sigma^+$ state, respectively. The method name y is composed of a standard acronym for the electronic structure method, which may be preceded by the number of correlated electrons if less than 21 electrons are correlated. We interpolate $V^{y/z}_{\mathrm{int},x}$ by fifth-order splines.

Across calculations, we use the augmented by diffuse functions correlation consistent basis sets from aug-cc-pVnZ, aug-cc-pCVnZ, aug-cc-pwCVnZ families, where $n \in \{T, Q, 5\}$.[1,2] We also use a basis set of six-zeta size (6Z). The aug-pCV6Z basis set for sodium comes from our previous work,[3] whereas the aug-pwCV6Z basis set for strontium has been obtained as a part of the current work (see section B for more details). In some calculations, we enrich the basis set by mid-bond functions. Although we have tested many of mid-bound functions, we report results only for two (see Table S1), which did not spoil the numerical stability of the calculations. The "mb2" has been created in an even-tempered manner and covers the entire range of exponents from i) mid-bond functions optimized for several systems by Show and Hill[4] ii) mid-bond functions proposed by Tao and Pen.[5] The "mb3" covers a range of exponents garnered by Show and Hill[4] for Rb_2 and Na_2 dimers. In order to reduce basis set superposition error, each $V^{y/z}_{\mathrm{int},x}$ was calculated as a difference between dimer and monomer energy using a dimer basis set.

Table S1: The exponents of mid-bond functions.

			mb2		
s	0.0327	0.0749	0.1716	0.3930	0.9001
p	0.0496	0.1303	0.3425	0.9000	
d	0.0546	0.1810	0.6000		
f	0.3				
			mb3		
s	0.03757	0.0779	0.1457		
p	0.05173	0.1793	0.2568		
d	0.0671	0.1482			

In the vicinity of the minimum, we obtained the ground-state interaction potential as:

$$V_{\rm int,gr} = V_{\rm int,gr}^{\rm CCSD(T)} + \delta V_{\rm int,gr}^{\rm CCSDT} + \delta V_{\rm int,gr}^{\rm CCSDT(Q)}. \tag{S1}$$

Leading terms in ground state interaction energy are obtained with spin-unrestricted open-shell coupled cluster singles, doubles, and noniterative triplets, CCSD(T),[6,7] using orbitals delivered by the restricted Hartree-Fock (ROHF) method. Although several tests were performed to check the convergence, the final calculations used the 6Z basis set enriched by the "mb2" mid-bond functions, thus

$$V_{\rm int,gr}^{\rm CCSD(T)} = V_{\rm int,gr}^{\rm CCSD(T)/6Z+mb2}. \tag{S2}$$

At this stage, we harness the MOLPRO 2022.1 package[8,9] to correlate all electrons, that are not replaced by ECP.

The leading contribution beyond CCSD(T) in coupled cluster expansions comes from iterative triple excitations.[10] We extrapolate this contribiution to complete-basis set limit using the two-point formula,[11] as:

$$\delta V_{\rm int,gr}^{\rm CCSDT/CBS(T,Q)} = \frac{4^3 \delta V_{\rm int,gr}^{\rm CCSDT/aug-cc-pwCVQZ} - 3^3 \delta V_{\rm int,gr}^{\rm CCSDT/aug-cc-pwCVTZ}}{4^3 - 3^3}, \tag{S3}$$

where

$$\delta V_{\rm int,gr}^{{\rm CCSDT}/z} = V_{\rm int,gr}^{{\rm CCSDT}/z} - V_{\rm int,gr}^{{\rm CCSD(T)}/z}. \tag{S4}$$

This part of calculations employs the CFOUR 2.1 software[12–14] to correlate 21 electrons.

Finally, we estimate the contribution from quadruple excitation, as:

$$\delta V_{\rm int,gr}^{\rm CCSDT(Q)/TZ} = V_{\rm int,gr}^{\rm 19e-CCSDT(Q)/aug-cc-pwCVTZ} - V_{\rm int,gr}^{\rm 19e-CCSDT/aug-cc-pwCVTZ}. \tag{S5}$$

At this stage, we reduce computational costs by excluding $1s$ electrons of sodium from CC

calculations. We obtain CCSDT(Q) in the B approximation,[15] as implemented in the MRCC 2018[16,17] code.

From here on, our focus shifts to calculating the excitation energy. We obtain the difference between ground and excited state interaction energies as:

$$\begin{aligned}\Delta V_{\text{int}} = &(V_{\text{int,exc}}^{\text{EOM-CCSD/6Z}} - V_{\text{int,gr}}^{\text{CCSD/6Z}}) \\ &+ (\delta V_{\text{int,exc}}^{\text{3e-CCSDT/CBS(T,Q)}} - \delta V_{\text{int,gr}}^{\text{3e-CCSDT/CBS(T,Q)}}) \\ &+ (\delta V_{\text{int,exc}}^{\text{coreCCSDT/TZ}} - \delta V_{\text{int,gr}}^{\text{coreCCSDT/TZ}}).\end{aligned} \tag{S6}$$

In order to properly localize the excited state with respect to the ground state we shift $V_{\text{int,exc}}$ by $E_{\text{exp,Sr}} = 14702.582\ \text{cm}^{-1}$, which is the experimental energy of the ^3P state (average over spin-orbit components) with respect to the ^1S state for Sr atom.[18]

The equation-of-motion coupled-cluster singles and doubles (EOM-CCSD)[19] provides a crucial part of the excitation energy. We use the orbitals delivered by the unrestricted Hartree-Fock method (UHF). Due to computational costs, here, we step away from using mid-bond functions in favour of the biggest available basis sets: 6Z. Here, all calculations utilize the CFOUR code.

The next step in the CC expansion would be the inclusion of the triple excitation, either as EOM-CCSDT,[20,21] or in any approximate form.[22–24] Disappointingly, none of the implementations available to us provided the numerical stability and efficiency sufficient to describe the open-shell NaSr molecule. Thus, we have to divide the triple excitation into two terms. The first, $\delta V_{\text{int,exc}}^{\text{3e-CCSDT/CBS(T,Q)}} - \delta V_{\text{int,gr}}^{\text{3e-CCSDT/CBS(T,Q)}}$, describes the triple excitation of valence electrons. We use the aug-cc-pVTZ and aug-cc-pVQZ basis sets. To be in the closest analogy to the ground state, we applied the equations analogical to Eq. (S3). Since the system has only three valence electrons, CCSDT and FCI become equivalent. Therefore, we use FCI[25,26] implementation available in MOLPRO.

The last included term is defined as:

$$\begin{aligned}\delta V_{\text{int},x}^{\text{coreCCSDT/TZ}} &= (V_{\text{int},x}^{\text{15e}-\text{CCSDT/TZ}} - V_{\text{int},x}^{\text{15e}-\text{CCSD/TZ}}) \\ &\quad - (V_{\text{int},x}^{\text{3e}-\text{CCSDT/TZ}} - V_{\text{int},x}^{\text{3e}-\text{CCSD/TZ}}).\end{aligned} \tag{S7}$$

Although $\delta V_{\text{int,exc}}^{\text{coreCCSDT/TZ}} - \delta V_{\text{int,gr}}^{\text{coreCCSDT/TZ}}$ describes the contribution of core and core-valence triple excitations to separation between electronic states, unfortunately, we are only able to obtain it with the aug-cc-pVTZ basis set. Additionally, we have to neglect the correlation of $1s2s$ electrons of Na and $4s$ of Sr. These shortcomings arise from the same problems that forced us to divide the triple contribution into two components. In this part, we utilize the FCI implementation of MOLPRO for all calculations where only valence electrons are correlated, and the LR-CCSDT implementation[27] of MRCC for calculations involving any core electron.

We introduce additional approximations for the parts of potential energy curves, which are distant from the minimum. Thus such approximations do not affect spectroscopic parameters, but allow us to elude from additional numerical problems. First, we assume that for interatomic separation shorter than 6.6 bohr, the contributions beyond leading CCSD(T) or EOM-CCSD are constant and equal to the values at 6.6 bohr. This approximation arises from the change in the nature of the Hartee-Fock determinant, which causes discontinuity at corrections for high excitation in the CC expansion. Additionaly, we use the long-range part (interatomic separation larger than 10 bohr) of ground state in the form:

$$s(V_{\text{int,gr}}^{\text{CCSD/6Z}} + \delta V_{\text{int,gr}}^{\text{valenceFCI/CBS(T,Q)}} + \delta V_{\text{int,gr}}^{\text{coreCCSDT/TZ}}), \tag{S8}$$

where $s = 1.00216$ is a scaling factor that guarantees the continuity of the potential energy curve at 10 bohr. The rest of the terms are the same as used for calculations of the gap between the ground end excited state. This approximation arises from our inability to reach a stable SCF solution when acting with a large basis set and mid-bond functions.

B New basis set for Sr

In order to get as close as possible to the complete basis set limit, we prepare a new basis set of the six-zeta size. We build our basis set by expanding the existing aug-cc-pwCV5Z-PP, which means that (i) we kept all s, p, d, f, g exponents from aug-cc-pwCV5Z-PP, (ii) we added exponents to s, p, d, f, g shells, (iii) we built from scratch h and i shells. All new exponents minimize the total CCSD(T) energy of the strontium dimer at 8.83 bohr. The optimization was performed in 10 steps. Only certain exponents were varied during the particular step of grid-based optimization. We discretized possible exponents by generating a grid between exponents from aug-cc-pwCV5Z-PP.

The first five steps incrementally improve the description of shells from s to g by adding two new exponents per shell. The exponents are determined by two-dimensional optimization of the CCSD(T) energy. During this process, only valence electrons are correlated. The h exponents from aug-cc-pwCV5Z-PP are used through all these five steps.

In the sixth step, we improve the h shell by replacing the three exponents with five freshly optimized exponents. At first, we find the two exponents for valence electrons minimizing the same energy as in earlier steps and augment the h shell by diffuse function in a typical even-tempered way. Thereafter, to improve the description of core electrons, we introduce two additional exponents that minimize the total energy produced by the CCSD(T) method, when the correlation of valence and $4p$ electrons is taken into account. In the final step, we build the i shell and add one exponent to the p shell. One valence and one core i exponents minimize the total energy obtained by the correlation of, respectively, 4 and 16 electrons with CCSD(T) method. Subsequently, we enrich the basis set by p and i exponents, that minimize the CCSD(T) energy of the lowest triplet state of the Sr_2 dimer. Using this new basis set we obtain -30.65987599 and -30.59300063 Hartree as the total CCSD(T) energy of Sr atom in ^1S and ^3P states, respectively.

Figure S1 shows the progress of different energies with the growth of the basis set. In panel (a), we see that the basis set optimization introduces only a minimal improvement in

the energy of valence electrons. Contrastingly, the description of core electrons remains far from complete (panel (b)). However, we may benefit from an error cancellation—the curves on panel (b) overlap. Differences between them do not exceed 15 cm^{-1}, as visualized on the panel (c).

The basis set is available in MOLPRO (file: aug-pwCV6Z.molpro) and CFOUR (file: aug-pwCV6Z.cfour) formats as separate supplementary text files.

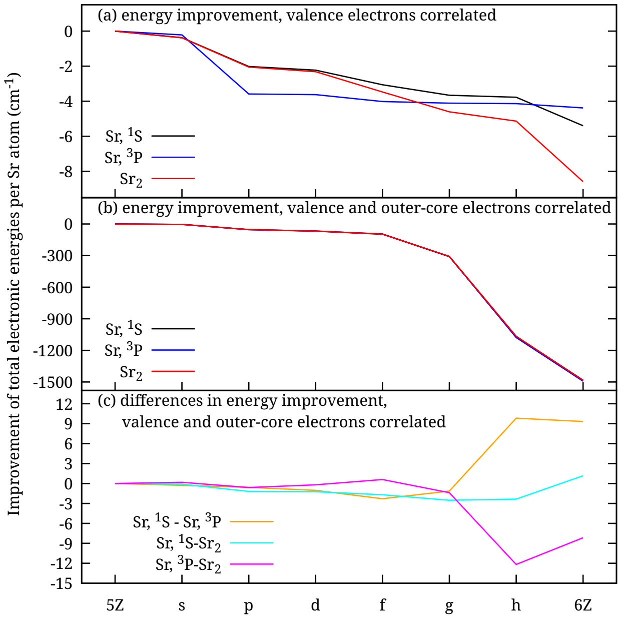

Figure S1: The changes in the total CCSD(T) energy with the expansion of basis set.

C Core electron creation and scalar relativistic effects in NaSr

Here, we investigate the influence of core electron correlation on the interaction energies in NaSr and the electronic-excitation energy in separated atoms. We compare the ECP-based (as described in Section 2.2 of the main publication and Section A) and all-electron calculations. In all-electron calculations we use (i) aug-cc-pwCVTZ-X2C basis set for Sr[2] (ii) aug-cc-pCVTZ-DK basis set for Na[1] (iii) one-electron variant of the spin-free exact two-

component theory, as implemented in CFOUR 2.1.[28] All calculations utilize a dimer basis set.

Table S2 collects energies for Na-Sr separation 7.5 bohr (≈ 4 Å). Generally, we observe a strengthening of the impact of electron correlation on spectroscopic parameters as we move from the inner to the outer core. The correlation of p electrons from the outer core is mandatory for semi-quantitative prediction of potential energy curves. We see that the application of small-core effective core potential alters interaction energies by just a few tens of cm^{-1}, which is not surprising as the correlation of $1s2s2p3s3p3d$ core electrons of strontium also alters interaction energies in NaSr by a similar amount. We found a comparable value for the strontium dimer.[29]

The lack of any core correlation increases the separation between ^2S (Na) + ^3P (Sr) and ^2P (Na) + ^1S (Sr) asymptotes from about 2300 cm^{-1} (in rough agreement with the experiment) to more than 3100 cm^{-1}. This may have profound influence on the calculated shape of the excited-state potential energy curve, due to the occurrence of avoided crossing between $2^2\Sigma^+$ (^2S of Na + ^3P of Sr) and $3^2\Sigma^+$ (^2P of Na + ^1S of Sr) at long-range region.

Table S2: The influence of core electron correlation on the CCSD interaction energies at interatomic separation equal 7.5 bohr (approx. 4 Å) and excitation energy of atoms. The atomic excitations energies are calculated in the dimer basis set.

	correlated electrons		interaction energy (cm^{-1})		excitation energy (cm^{-1})	
	Na	Sr	$X(1)^2\Sigma^+$	$B(2)^2\Sigma^+$	$^2P -^2 S$, Na	$^3P -^1 S$, Sr
X2C-1e	1s2s2p3s	1s2s2p3s3p3d4s4p5s	-1259.3	-5663.2	16826.8	14517.9
	1s2s2p3s	2s2p3s3p3d4s4p5s	-1259.3	-5663.3	16826.8	14518.0
	1s2s2p3s	3s3p3d4s4p5s	-1259.3	-5663.8	16826.8	14518.4
	2s2p3s	3s3p3d4s4p5s	-1259.6	-5663.9	16834.5	14518.4
	2s2p3s	3d4s4p5s	-1261.1	-5673.7	16834.5	14523.4
	2s2p3s	4s4p5s	-1263.3	-5681.4	16834.5	14519.1
	2p3s	4s4p5s	-1274.3	-5698.6	16963.3	14519.1
	2p3s	4p5s	-1282.7	-5735.9	16963.3	14507.3
	3s	4p5s	-1315.2	-5991.2	16000.2	14507.3
	3s	5s	-1510.8	-5508.2	16000.2	12831.6
	1s2s2p3s	4s4p5s	-1263.0	-5681.3	16826.8	14519.1
ECP	1s2s2p3s	4s4p5s	-1255.8	-5700.7	16764.4	14546.4
	2s2p3s	4s4p5s	-1256.2	-5700.8	16772.1	14546.4
	2p3s	4s4p5s	-1267.1	-5716.7	16900.0	14546.4
	2p3s	4p5s	-1289.3	-5731.1	16900.0	14472.0
	3s	4p5s	-1321.9	-5988.7	15944.8	14472.0
	3s	5s	-1508.1	-5513.8	15944.8	12838.6

D Convergence of the composite method

Leading contributions to the interaction energy converge with basis set similarly for both the ground and the excited state. Both potentials systematically deepen with an increasing basis set cardinal number (see Table S3). An increase in the size of mid-bond functions also deepens both potentials. Surprisingly, the interaction energy is sensitive to the reference function. However, this will be correct at higher correlation levels as the difference between UHF- and ROHF-based calculations decreases when we include higher and higher excitations. At the CCSDT level, the choice of reference wavefunction alters interaction energy by roughly 10 cm^{-1} (see Table S4). We observe a moderate impact of the choice of the basis set family on the energy. The mid-bond functions deepen both potentials, but increasing the basis set cardinal number has a more profound effect on the potentials. Overall, we converge the leading contribution to the interaction energy with accuracy of the order of several cm^{-1}.

We estimate the precision of $V_{\mathrm{int,gr}}$ in semi-quantitive way. All numbers given in this paragraph refer to minima on the ground state potential energy curve. First, we estimate uncertainty of $V_{\mathrm{int,gr}}^{\mathrm{CCSD(T)}}$ as arithmetic mean of

$$\left| V_{\mathrm{int,gr}}^{\mathrm{CCSD(T)/6Z+mb2}} - V_{\mathrm{int,gr}}^{\mathrm{CCSD(T)/6Z}} \right| \tag{S9}$$

and

$$\left| V_{\mathrm{int,gr}}^{\mathrm{CCSD(T)/6Z+mb2}} - V_{\mathrm{int,gr}}^{\mathrm{CCSD(T)/5Z+mb2}} \right| . \tag{S10}$$

It comes to 5 cm^{-1}. The full triples correction, $\delta V_{\mathrm{int,gr}}^{\mathrm{CCSDT}}$ amount -66 cm^{-1} and -59 cm^{-1}, respectively, for aug-cc-pwCVTZ and aug-cc-pwCVQZ basis set. We estimate uncertentity of this term as:

$$\delta V_{\mathrm{int,gr}}^{\mathrm{CCSDT/CBS(T,Q)}} - \delta V_{\mathrm{int,gr}}^{\mathrm{CCSDT/aug-cc-pwCVQZ}} \approx 6\ \mathrm{cm}^{-1}. \tag{S11}$$

The perturbative quadruple excitations alter the interaction energy by -34 cm^{-1}, which

is of a similar order of magnitude as in the case of full triple excitations. We assume that calculations in the triple-zeta basis set recover a similar fraction of full triple and perturbative quadruple contributions. Thus, we estimate the uncertainty of $\delta V_{\mathrm{int,gr}}^{\mathrm{CCSDT(Q)}}$ as 7 cm^{-1}. We assume that the higher excitations in CC expansion may change potential by the half of $\delta V_{\mathrm{int,gr}}^{\mathrm{CCSDT(Q)}}$, roughly 17 cm^{-1}. The relativistic contribution and ECP may introduce changes in interaction energies about 4 cm^{-1} (see section C). The neglected terms arise from (i) going beyond Born-Oppenheimer approximation, (ii) spin-related part of the Dirac-Coulomb-Breit Hamiltonian, and (iii) quantum electrodynamics. However, each of them should be much lower than any other source of uncertainty in our calculations. We assume that negleted terms in Hamiltonian sum up to 1 cm^{-1}. Finally, uncertainty of $V_{\mathrm{int,gr}}$ reaches 40 cm^{-1}.

Let us focus on the excited state potential energy curve. First, we analyze the uncertainty of ΔV_{int}. The leading contribution is predicted with surprisingly high precision, as the ground and excited state converge parallel with the basis set size. The difference between CCSD-based excitation energy predicted with 5-zeta and 6-zeta basis sets exceeds 3 cm^{-1} only at the repulsive wall. Similarly, the CCSDT correction to excitation energy seems to be converged with a precision of about 2 cm^{-1}, as long as we consider only valence electrons. The relativistic contribution and ECP may introduce changes in interaction energies about 38 cm^{-1} (see section C). The most problematic term is the contribution of the higher excitations involving core electrons. As described in A, we provide only a rough estimation of this term. Simultaneous, the core correlation may greatly alter the separation between excited states, potentially modifying avoided crossing, and thus shifting the potential energy curve. In this situation, we assume that the uncertainty of this term is 50% (30 cm^{-1}). So, we expect that we calculate the differences between the spin-orbit-free interaction potential of the ground and the excited state with uncertainty of the order of 73 cm^{-1}. Nevertheless, the spin-orbit interaction is important in this system. The complexity may arise from the crossing with $1^2\Pi$ state (Fig. S2), which happens in the vicinity of minima of $1^2\Sigma^+$. The spin-orbit interaction may mix the $\Omega = 1/2$ components of $1^2\Pi$ and $1^2\Sigma^+$ states. However,

our rough estimation suggests that spin-orbit interaction changes the excitation energy in the vicinity of minima by several cm^{-1}, but greatly modifies the long-range part of the potential. This is not surprising, as the experimentally measured separation $^3P_0 - ^3P_2$ is 581 cm^{-1}.[18] Thus, we see that spin-orbit interaction may change the depth of potential by hundreds of cm^{-1}. Anyway, the predicted by CCSD separation $^2S - ^3P$ is lower by 123 cm^{-1} than $E_{\mathrm{exc,Sr}}$. The correction on the full triples lowers the singlet-triplet gap by 30 cm^{-1} to 14550 cm^{-1}. Overall, we expect that the T_e may be overestimated by a few hundred cm^{-1}. However, the shape of the potential in the vicinity of minima should be predicted with precision only slightly worse than in the case of the ground state, due to cancellation of errors.

Table S3: The interaction energies at interatomic separation equal 7.5 bohr (approx. 4 Å) and the excitation energy of Sr predicted by different methods and basis sets. The atomic excitation energies are calculated in the dimer basis set.

Na	Sr	mb	SCF	$X^2\Sigma^+$		$2^2\Sigma^+$	^3P
				CCSD(T)	CCSD	EOM-CCSD	EOM-CCSD
aug-cc-pwCVTZ	aug-cc-pwCVTZ-PP	-	UHF	-1401.0	-1255.4	-5698.6	14546.7
aug-cc-pwCVQZ	aug-cc-pwCVQZ-PP	-	UHF	-1465.5	-1306.7	-5749.7	14595.6
aug-cc-pwCV5Z	aug-cc-pwCV5Z-PP	-	UHF	-1494.70	-1332.6	-5762.5	14582.5
aug-cc-pCVTZ	aug-cc-pCVTZ-PP	-	UHF	-1411.5	-1267.6	-5689.9	14444.5
aug-cc-pCVQZ	aug-cc-pCVQZ-PP	-	UHF	-1474.5	-1317.1	-5731.0	14536.8
aug-cc-pCV5Z	aug-cc-pCV5Z-PP	-	UHF	-1496.3	-1335.7	-5758.5	14561.7
aug-cc-pCVTZ	aug-cc-pwCVTZ-PP	-	UHF	-1397.2	-1257.1	-5736.3	14546.6
aug-cc-pCVQZ	aug-cc-pwCVQZ-PP	-	UHF	-1465.5	-1306.7	-5749.7	14595.6
aug-cc-pCV5Z	aug-cc-pwCV5Z-PP	-	UHF	-1492.9	-1331.7	-5765.0	14582.5
aug-pCV6Z	aug-pwCV6Z-PP	-	UHF	-1504.0	-1341.5	**-5771.2**	14579.6
aug-cc-pwCVQZ	aug-cc-pwCVQZ-PP	mb3	UHF		-1318.8	-5762.2	14595.8
aug-cc-pwCV5Z	aug-cc-pwCV5Z-PP	mb3	UHF		-1335.5	-5766.2	14583.1
aug-cc-pwCVQZ	aug-cc-pwCVQZ-PP	mb2	UHF		-1326.9	-5769.6	14595.3
aug-cc-pwCV5Z	aug-cc-pwCV5Z-PP	mb2	UHF		-1339.0	-5769.5	14582.8
aug-pCV6Z	aug-pwCV6Z-PP	mb2	UHF		-1342.0		14579.7
aug-cc-pwCVTZ	aug-cc-pwCVTZ-PP	-	ROHF	-1426.2	-1170.1		
aug-cc-pwCVQZ	aug-cc-pwCVQZ-PP	-	ROHF	-1494.1	-1220.4		
aug-cc-pwCV5Z	aug-cc-pwCV5Z-PP	-	ROHF	-1523.7	-1244.0		
aug-cc-pCVTZ	aug-cc-pwCVTZ-PP	-	ROHF	-1420.7	-1169.1		
aug-cc-pCVQZ	aug-cc-pwCVQZ-PP	-	ROHF	-1490.1	-1218.6		
aug-cc-pCV5Z	aug-cc-pwCV5Z-PP	-	ROHF	-1521.8	-1242.8		
aug-pCV6Z	aug-pwCV6Z-PP	-	ROHF	-1533.5	-1251.9		
aug-cc-pCV5Z	aug-cc-pwCV5Z-PP	mb3	ROHF	-1525.7	-1246.0		
aug-pCV6Z	aug-pwCV6Z-PP	mb3	ROHF	-1534.4	-1252.5		
aug-cc-pCV5Z	aug-cc-pwCV5Z-PP	mb2	ROHF	-1529.6	-1249.2		
aug-pCV6Z	aug-pwCV6Z-PP	mb2	ROHF	**-1536.5**	-1254.3		

Table S4: The CCSDT interaction energies of $X^2\Sigma^+$ state (cm^{-1}).

Na	Sr	SCF	$X^2\Sigma^+$
aug-cc-pwCVTZ	aug-cc-pwCVTZ-PP	UHF	-1501.5
aug-cc-pCVTZ	aug-cc-pCVTZ-PP	UHF	-1510.0
aug-cc-pCVTZ	aug-cc-pwCVTZ-PP	UHF	-1496.8
aug-cc-pwCVTZ	aug-cc-pwCVTZ-PP	ROHF	-1492.3
aug-cc-pCVTZ	aug-cc-pCVTZ-PP	ROHF	-1499.9
aug-cc-pCVTZ	aug-cc-pwCVTZ-PP	ROHF	-1487.5
aug-cc-pwCVQZ	aug-cc-pwCVQZ-PP	ROHF	-1552.5

E Additional Figures

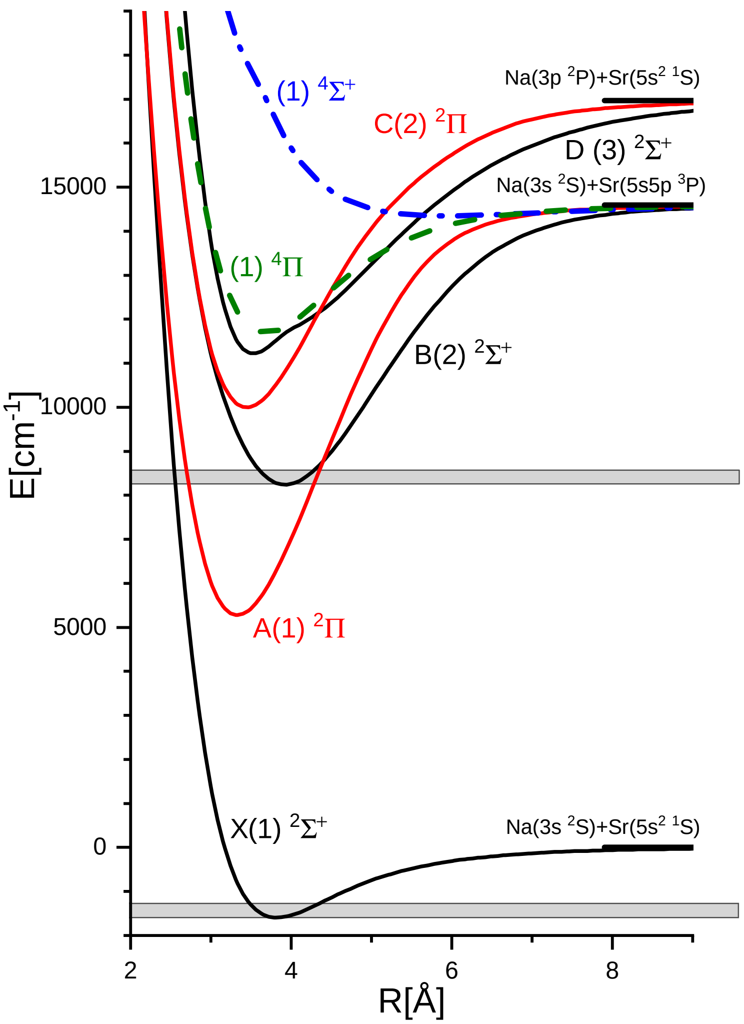

Figure S2: PECs of the ground state and excited states of NaSr up to the Na(3p ^2P)+Sr(5s^2 ^1P) dissociation limit calculated with the (ECP+CPP)FCI method. The grey-shaded area corresponds to the energy ranges studied experimentally.

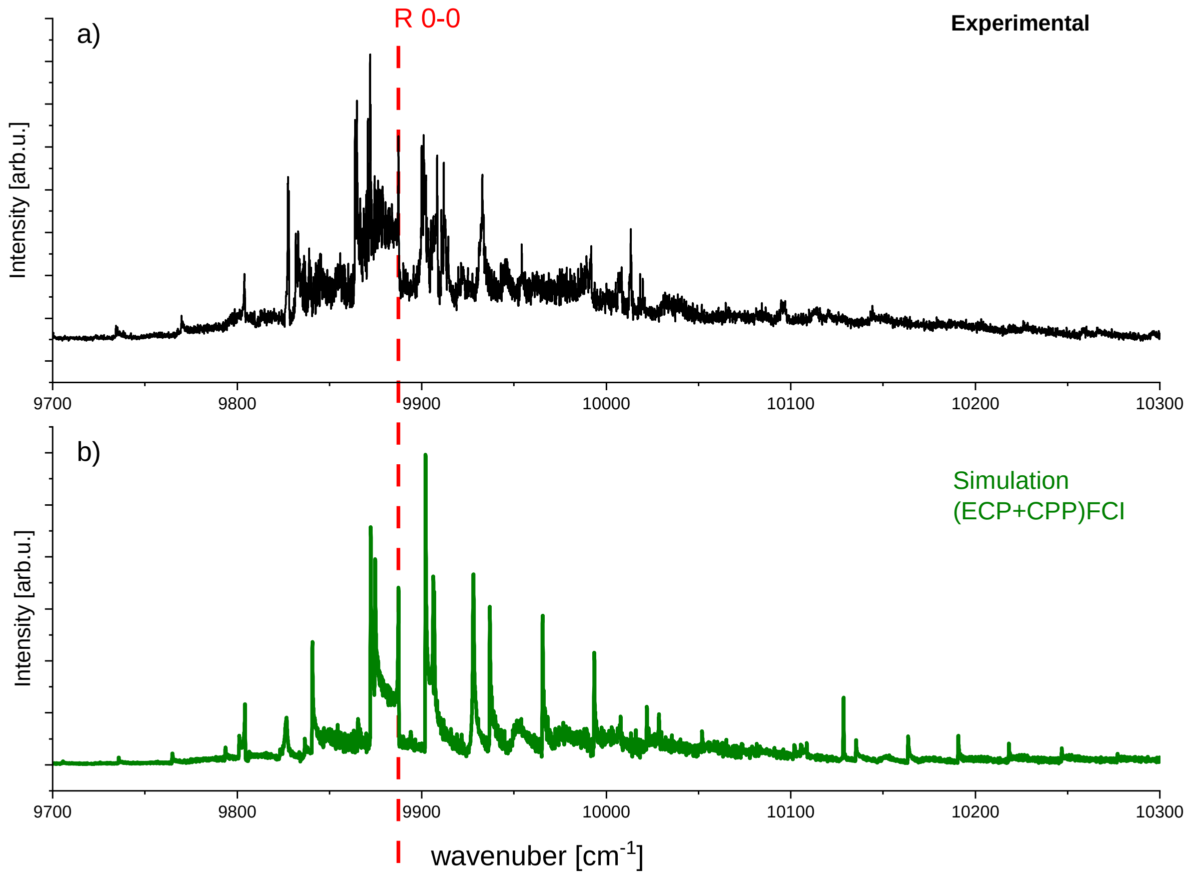

Figure S3: Comparison of the experimental thermoluminescence spectrum (a) with the simulated spectrum based on theoretical curves. The theoretical spectrum was shifted so that the onset of the 0-0 band coincides with its position in the experimental spectrum; (b) spectrum resulting from the (ECP+CPP)FCI calculations (this work) shifted by $\approx +26\,\mathrm{cm}^{-1}$

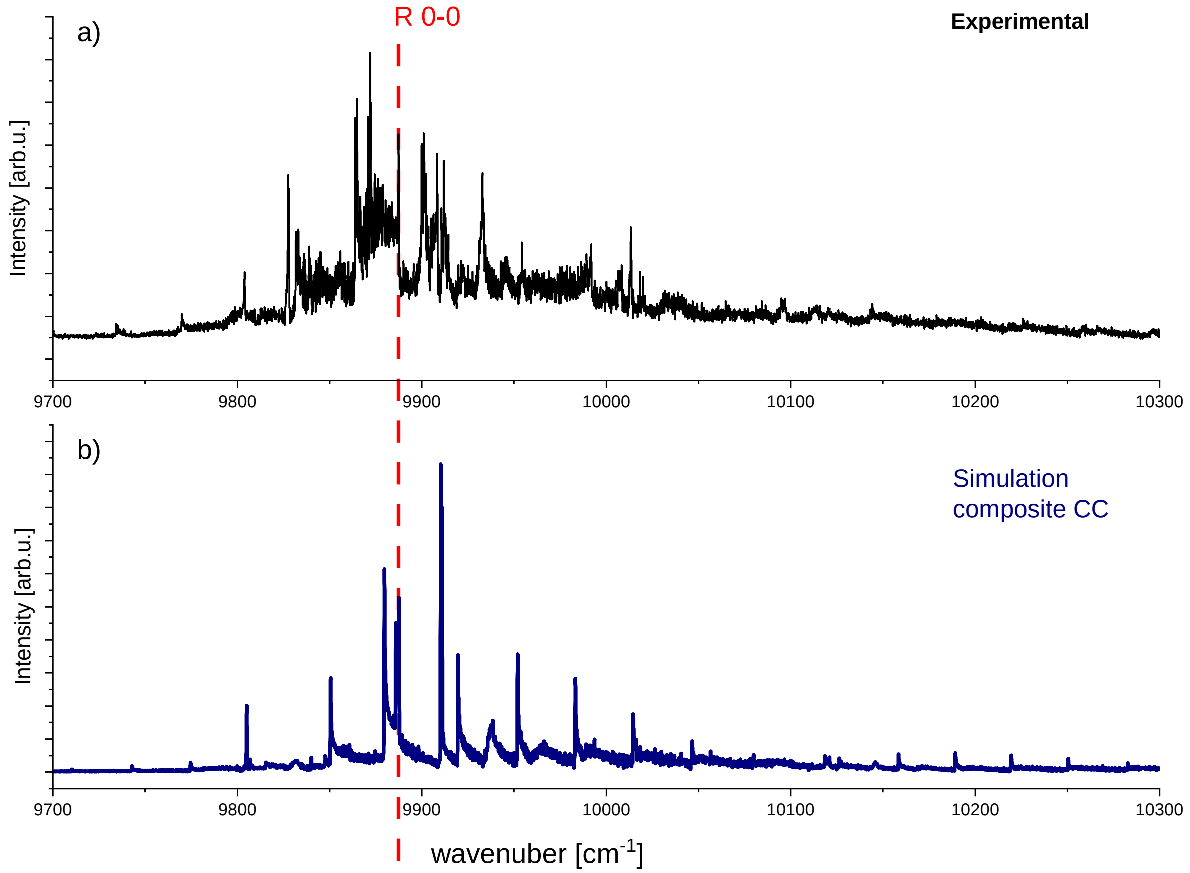

Figure S4: Comparison of the experimental thermoluminescence spectrum (a) with the simulated spectrum based on theoretical curves. The theoretical spectrum was shifted so that the onset of the 0-0 band coincides with its position in the experimental spectrum; (b) spectrum resulting from the composite CC calculations (this work) shifted by $\approx -251\ \mathrm{cm}^{-1}$

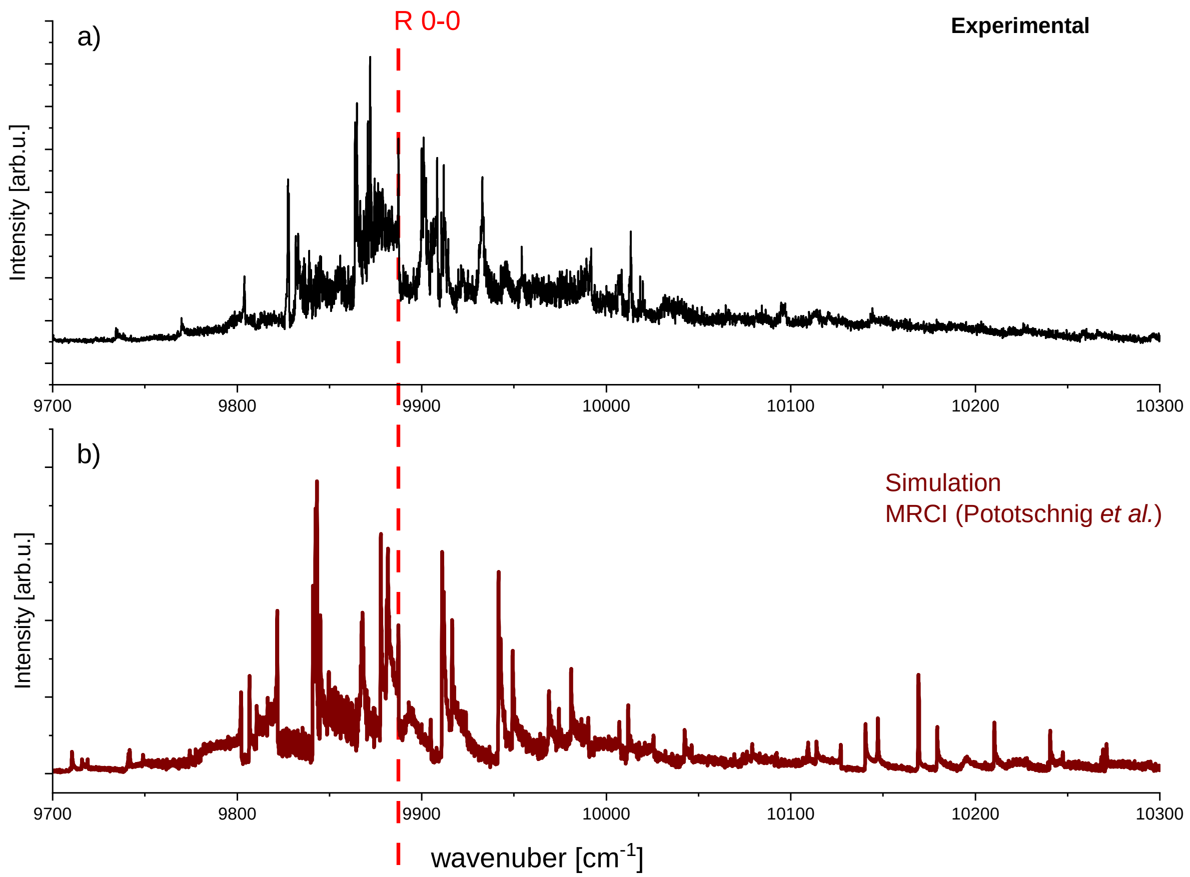

Figure S5: Comparison of the experimental thermoluminescence spectrum (a) with the simulated spectrum based on theoretical curves. The theoretical spectrum was shifted so that the onset of the 0-0 band coincides with its position in the experimental spectrum; (b) spectrum resulting from the MRCI calculations (Pototschnig et al.)[30] shifted by $\approx +71\,\mathrm{cm}^{-1}$

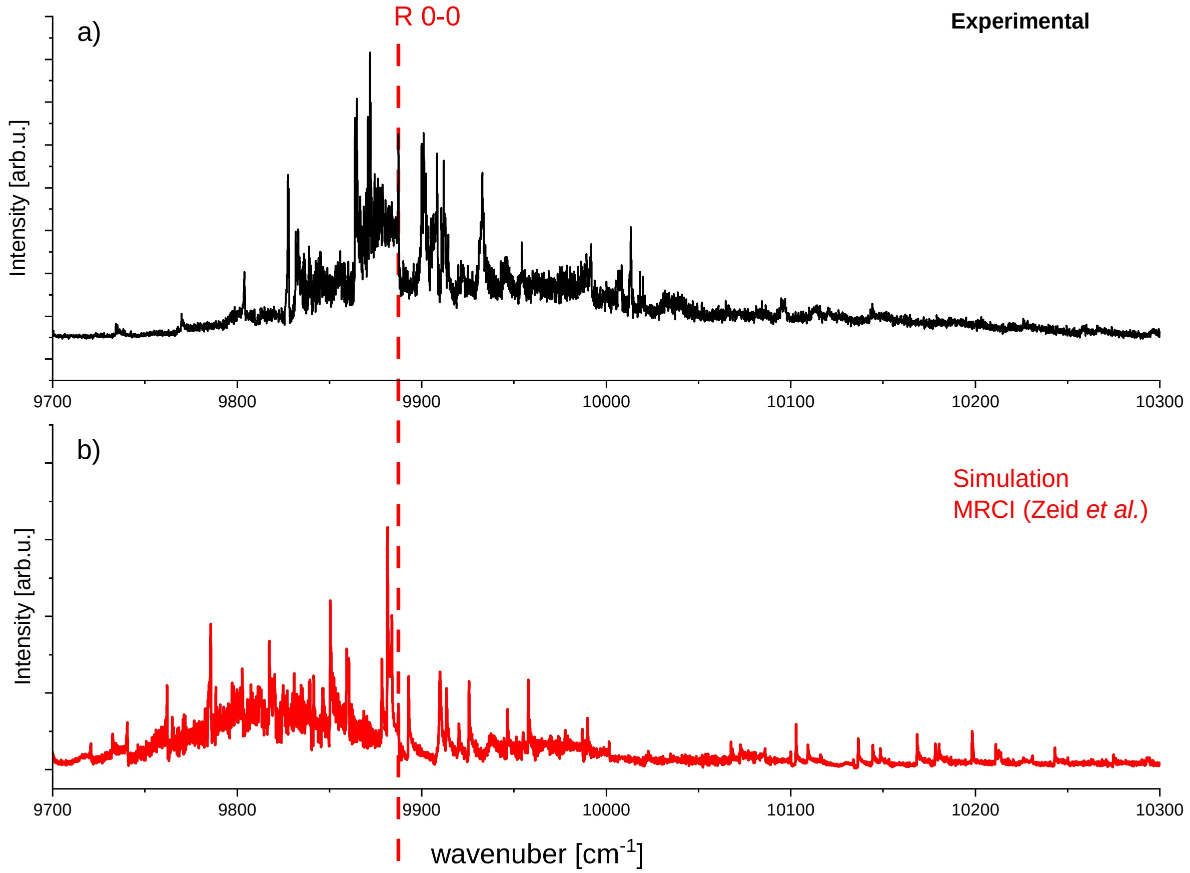

Figure S6: Comparison of the experimental thermoluminescence spectrum (a) with the simulated spectrum based on theoretical curves. The theoretical spectrum was shifted so that the onset of the 0-0 band coincides with its position in the experimental spectrum; (b) spectrum resulting from the MRCI calculations (Zaid et al.)[31] shifted by $\approx -687\,\mathrm{cm}^{-1}$

F PECs calculated by the CCC method

Table S5: Potential energy curves of $X(1)^2\Sigma^+$ and $B(2)^2\Sigma^+$ states calculated using the Composite Coupled-Cluster method. Distances are in Angstrom, energies in cm^{-1} units.

R	$X(1)^2\Sigma^+$	$B(2)^2\Sigma^+$
2.60	9851.4639202	18659.5684139
2.65	8922.9816408	17930.8986843
2.70	7920.7614722	17135.7442080
2.75	6992.5326963	16406.4054849
2.80	6138.3024684	15741.9090068
2.85	5358.0787615	15141.9278510
2.90	4651.8664745	14606.6752328
2.95	4016.3071784	14130.6122091
3.00	3444.2387374	13706.8526887
3.03	3131.0403726	13474.1129071
3.06	2838.0041388	13254.0957313
3.09	2564.6745073	13046.0449652
3.12	2311.0555689	12849.6234916
3.15	2075.7836569	12663.1239632
3.18	1857.0829464	12484.2333891
3.21	1654.9442912	12311.9221789
3.24	1469.1785279	12147.2562001
3.27	1298.0583216	11988.7717704
3.30	1141.1613318	11836.0939113
3.33	997.3840805	11689.0297703
3.36	864.0601619	11545.7733347
3.39	741.0758587	11407.2190005

3.42	628.4630198	11274.3931793
3.45	526.9917583	11149.0430644
3.48	437.0595507	11032.5263260
3.51	361.8823805	10928.8751326
3.54	296.8499133	10834.4783936
3.57	238.7163036	10746.5934801
3.60	187.4847125	10665.2900394
3.63	143.3144743	10590.4892956
3.66	105.9794728	10523.0432730
3.69	75.3898003	10463.2740213
3.72	50.5911006	10410.5598732
3.75	31.2235057	10364.9900419
3.78	16.8166340	10326.2631996
3.81	7.0837270	10294.1301937
3.84	1.6365461	10268.4117095
3.87	0.0025687	10249.0665255
3.90	1.9779704	10235.8044021
3.93	7.1762878	10228.3944576
3.96	15.3659966	10226.7230035
3.99	26.1715997	10230.5064253
4.02	39.4341489	10239.7117294
4.05	54.7609153	10254.0432882
4.08	71.9934278	10273.2191185
4.11	90.9227497	10296.9073125
4.14	111.2850507	10324.9590498
4.17	132.9881867	10357.5749420
4.20	155.9118490	10394.3924552

4.23	179.6424996	10434.9759742
4.26	204.1452328	10479.3038960
4.29	229.4167746	10527.2983099
4.32	255.2139669	10578.6636200
4.35	281.3812824	10633.1844454
4.38	307.9191261	10690.7969836
4.41	334.7707219	10751.3607406
4.44	361.6988158	10814.5542348
4.47	388.6770252	10880.2553216
4.50	415.7046202	10948.3739539
4.53	442.6562011	11018.6996830
4.56	469.4333408	11091.0200397
4.59	496.0364340	11165.2373808
4.62	522.4470706	11241.2197905
4.65	548.5715515	11318.7645425
4.68	574.3908186	11397.7516247
4.71	599.8982958	11478.0554569
4.74	625.0441914	11559.5094741
4.77	649.7804755	11641.9548282
4.80	674.1101315	11725.2887029
4.83	698.0283506	11809.3867962
4.86	721.4965503	11894.0965211
4.89	744.5079922	11979.2917071
4.92	767.0624960	12064.8645570
4.95	789.1613870	12150.7099580
4.98	810.8058390	12236.7151172
5.01	831.9959939	12322.7627136

5.04	852.7273378	12408.7630899
5.07	872.9634127	12494.5766974
5.10	892.6963587	12580.0673632
5.13	911.9258845	12665.1412743
5.16	930.7607562	12749.8249202
5.19	949.3584386	12834.1807321
5.22	967.7203966	12918.1112152
5.25	985.8496123	13001.5352619
5.28	1003.7779444	13084.3934068
5.31	1020.4880120	13165.6724724
5.34	1036.2071223	13245.4393104
5.37	1051.5614726	13324.1686486
5.40	1066.5558182	13401.7810493
5.43	1081.1928174	13478.1749750
5.46	1095.4807538	13553.3161301
5.49	1109.4255719	13627.1414676
5.52	1123.0342405	13699.5765875
5.55	1136.3124023	13770.5676248
5.58	1149.2648731	13840.0730308
5.61	1161.8974281	13908.0402111
5.64	1174.2236265	13974.2914465
5.67	1186.2443634	14038.9222233
5.70	1197.9651850	14101.9192441
5.73	1209.3912017	14163.2749583
5.76	1220.5283540	14222.9758695
5.79	1231.3836541	14281.0034745
5.82	1241.9634420	14337.3452442

5.85	1252.2814068	14391.7849615
5.88	1262.3349368	14444.5768040
5.91	1272.1274218	14495.7995486
5.94	1281.6582247	14545.6231638
5.97	1290.9309384	14594.1602112
6.00	1299.9581716	14641.3210297
6.03	1308.7475333	14687.1244290
6.06	1317.3080666	14731.5718825
6.09	1325.6453287	14774.7041180
6.12	1333.7659858	14816.5489106
6.15	1341.6785595	14857.1180024
6.18	1349.3913504	14896.4253476
6.21	1356.9079502	14934.5272071
6.24	1364.2343431	14971.4595157
6.27	1371.3764521	15007.2578503
6.30	1378.3402373	15041.9578856
6.33	1385.1317361	15075.5957496
6.36	1391.7644987	15108.7226819
6.39	1398.2518199	15142.0448942
6.42	1404.5811673	15174.6249202
6.45	1410.7549404	15206.4988868
6.48	1416.7755133	15237.7041212
6.51	1422.6453049	15268.2781162
6.54	1428.3667534	15298.2583998
6.57	1433.9422712	15327.6823024
6.60	1439.3743489	15356.5875050
6.63	1444.6654113	15385.0112836

6.66	1449.8179745	15412.9913445
6.69	1454.8345071	15440.5650866
6.72	1459.7174883	15467.7699094
6.75	1464.4694644	15494.6435358
6.78	1469.0929204	15521.2232945
6.81	1473.5904093	15547.5468549
6.84	1477.9644527	15573.6516543
6.87	1482.2175131	15599.5699591
6.90	1486.3210652	15623.3446217
6.93	1490.2891918	15645.5939525
6.96	1494.1380053	15667.1516581
6.99	1497.8704400	15688.0339693
7.02	1501.4895002	15708.2573658
7.05	1504.9981506	15727.8380687
7.08	1508.3993628	15746.7923091
7.11	1511.6961546	15765.1365444
7.14	1514.8915002	15782.8869607
7.17	1517.9884187	15800.0599648
7.20	1520.9899100	15816.6718309
7.23	1523.8989854	15832.7388720
7.26	1526.7186733	15848.2774697
7.29	1529.4519789	15863.3038554
7.32	1532.1019509	15877.8344773
7.35	1534.6715973	15891.8855389
7.38	1537.1639766	15905.4734988
7.41	1539.5840191	15918.6232943
7.44	1541.9840329	15931.5757876

7.47	1544.3140875	15944.1058834
7.50	1546.5769582	15956.2286605
7.53	1548.7747039	15967.9559430
7.56	1550.9093572	15979.2993972
7.59	1552.9829653	15990.2707557
7.62	1554.9975873	16000.8817966
7.65	1556.9552631	16011.1441840
7.68	1558.8580652	16021.0697411
7.71	1560.7080349	16030.6701145
7.74	1562.5072497	16039.9571281
7.77	1564.2577602	16048.9424539
7.80	1565.9616411	16057.6378787
7.83	1567.6209596	16066.0551382
7.86	1569.2377777	16074.2059346
7.89	1570.8141810	16082.1020779
7.92	1572.3522754	16089.7555127
7.95	1573.9103691	16097.5182783
7.98	1575.5088927	16105.5036069
8.01	1577.0648736	16113.2065495
8.04	1578.5793250	16120.6348885
8.07	1580.0530864	16127.7953727
8.10	1581.4870257	16134.6948822
8.13	1582.8819894	16141.3401870
8.16	1584.2388419	16147.7381373
8.19	1585.5584408	16153.8955431
8.22	1586.8416448	16159.8192159
8.25	1588.0893219	16165.5160028

8.28	1589.3023273	16170.9926891
8.31	1590.4815356	16176.2561407
8.34	1591.6278075	16181.3131584
8.37	1592.7420062	16186.1705490
8.40	1593.8250096	16190.8351789
8.43	1594.8776774	16195.3138318
8.46	1595.9010778	16199.6149914
8.49	1596.9342264	16204.0722730
8.52	1597.9533940	16208.4817380
8.55	1598.9476604	16212.7491884
8.58	1599.9174279	16216.8774182
8.61	1600.8631025	16220.8692187
8.64	1601.7850804	16224.7273351
8.67	1602.6837719	16228.4545698
8.70	1603.5595770	16232.0536798
8.73	1604.4128979	16235.5274269
8.76	1605.2441462	16238.8786100
8.79	1606.0537218	16242.1099781
8.82	1606.8420377	16245.2243294
8.85	1607.6094964	16248.2244199
8.88	1608.3565090	16251.1130351
8.91	1609.0834840	16253.8929495
8.94	1609.7908286	16256.5669310
8.97	1610.4789568	16259.1377704
9.00	1611.1482739	16261.6082248
9.10	1613.2487824	16269.1538239
9.15	1614.2271511	16272.5521075

9.20	1615.1601483	16275.7178674
9.25	1616.0496899	16278.6639608
9.30	1616.8976950	16281.4032482
9.35	1617.7060908	16283.9486077
9.40	1618.4768004	16286.3128962
9.45	1619.2117550	16288.5089888
9.50	1619.9128844	16290.5497420
9.55	1620.6222193	16292.7598193
9.60	1621.3417771	16295.1527056
9.65	1622.0316980	16297.4162677
9.70	1622.6928430	16299.5549819
9.75	1623.3260735	16301.5733092
9.80	1623.9322506	16303.4757047
9.85	1624.5122376	16305.2666264
9.90	1625.0669027	16306.9505422
9.95	1625.5971110	16308.5319066
10.00	1626.1037329	16310.0151867
10.05	1626.5876386	16311.4048450
10.10	1627.0496986	16312.7053412
10.15	1627.4907884	16313.9211466
10.20	1627.9117807	16315.0567222
10.25	1628.3135517	16316.1165353
10.30	1628.6969787	16317.1050538
10.35	1629.0629390	16318.0267429
10.40	1629.4123113	16318.8860702
10.45	1629.7459768	16319.6875071
10.50	1630.0648150	16320.4355189

10.55	1630.3697089	16321.1345771
10.60	1630.6751426	16321.8676158
10.65	1630.9959044	16322.7193196
10.70	1631.3046409	16323.5312608
10.75	1631.6016790	16324.3046934
10.80	1631.8873512	16325.0408972
10.85	1632.1619913	16325.7411528
10.90	1632.4259320	16326.4067380
10.95	1632.6795077	16327.0389331
11.00	1632.9230542	16327.6390201
11.05	1633.1569059	16328.2082776
11.10	1633.3813998	16328.7479885
11.15	1633.5968720	16329.2594334
11.20	1633.8036589	16329.7438925
11.25	1634.0020993	16330.2026498
11.30	1634.1925302	16330.6369857
11.35	1634.3752904	16331.0481832
11.40	1634.5507189	16331.4375252
11.45	1634.7191544	16331.8062932
11.50	1634.8809365	16332.1557706
11.55	1635.0364058	16332.4872412
11.60	1635.1859020	16332.8019869
11.65	1635.3323112	16333.1120684
11.70	1635.4862989	16333.4624808
11.75	1635.6351286	16333.7991395
11.80	1635.7789013	16334.1223097
11.85	1635.9177506	16334.4323949

11.90	1636.0518114	16334.7298013
11.95	1636.1812187	16335.0149346
12.00	1636.3061070	16335.2881994
12.20	1636.7631803	16336.2706951
12.30	1636.9681311	16336.7012890
12.40	1637.1588115	16337.0957809
12.50	1637.3363158	16337.4574228
12.60	1637.5017403	16337.7894678
12.70	1637.6561686	16338.0951227
12.80	1637.8137782	16338.4190338
12.90	1637.9619253	16338.7209443
13.00	1638.1009910	16339.0018204
13.10	1638.2313764	16339.2626869
13.20	1638.3534840	16339.5045689
13.30	1638.4677180	16339.7284922
13.40	1638.5744836	16339.9354839
13.50	1638.6741880	16340.1265716
13.60	1638.7672391	16340.3027833
13.70	1638.8540458	16340.4651479
13.80	1638.9393115	16340.6259652
13.90	1639.0257955	16340.7924301
14.00	1639.1078451	16340.9496505
14.10	1639.1856003	16341.0979413
14.20	1639.2592022	16341.2376181
14.30	1639.3287926	16341.3689967
14.40	1639.3945138	16341.4923931
14.50	1639.4565088	16341.6081237

14.60	1639.5149214	16341.7165057
14.70	1639.5698956	16341.8178558
14.80	1639.6215760	16341.9124915
14.90	1639.6701080	16342.0007304
15.00	1639.7156372	16342.0828905

Table S5: Potential energy curves of $X(1)^2\Sigma^+$ and $B(2)^2\Sigma^+$ states calculated using the Composite Coupled-Cluster method. Distances are in Angstrom, energies in cm^{-1} units.

G PECs and TDMs calculated by the (ECP+CPP)FCI method

Table S6: Potential energy curves of $^2\Sigma^+$ states calculated using the (ECP+CPP)FCI method. Distances in atomic units, energies in Hartree.

R	$1^2\Sigma^+$ Na(3s)+Sr(5s^2 ^{1}S)	$2^2\Sigma^+$ Na(3s)+Sr(5s5p ^{3}P)	$3^2\Sigma^+$ Na(3p)+Sr(5s^2 ^{1}S)	$4^2\Sigma^+$ Na(3s)+Sr(5s4d ^{3}D)	$5^2\Sigma^+$ Na(3s)+Sr(5s4d ^{1}D)	$6^2\Sigma^+$ Na(3s)+Sr(5s5p ^{1}P)
3.0	-0.621201867617	-0.581149996449	-0.537890516180	-0.530383805245	-0.525186305940	-0.522265083887
3.3	-0.648646106401	-0.612632746328	-0.568991406853	-0.558860642725	-0.554926423919	-0.552573257469
3.6	-0.675185345036	-0.641913454852	-0.598523360885	-0.586536171466	-0.582091710717	-0.579653237436
3.9	-0.700724538590	-0.668459548830	-0.627786581451	-0.613647529269	-0.605885003894	-0.604029467270
4.2	-0.724760186216	-0.691735536515	-0.656064529400	-0.640513651194	-0.627516485000	-0.626063829006
4.5	-0.746344094918	-0.711255674230	-0.681415130327	-0.665310033874	-0.649958594091	-0.643708451772
4.8	-0.764637365408	-0.726859788737	-0.702717747628	-0.686459053064	-0.669326749325	-0.659602606971
5.1	-0.779303624039	-0.738758252753	-0.719749930008	-0.703690243755	-0.684770619201	-0.674717767612
5.4	-0.790488274342	-0.747432291629	-0.732780977083	-0.717289980175	-0.696414485439	-0.686111810931
5.7	-0.798621348596	-0.753561456983	-0.742229301051	-0.727719282519	-0.704758285792	-0.694288946853
6.0	-0.804240314425	-0.757953157798	-0.748450416446	-0.735473593382	-0.710402259180	-0.699858949923
6.3	-0.807881766011	-0.761298583170	-0.751811755593	-0.741018933551	-0.713919241975	-0.703397482103
6.6	-0.810029182916	-0.763835444508	-0.752967424284	-0.744761380268	-0.715805133912	-0.705422848832
6.9	-0.811093689316	-0.765533362444	-0.752693916581	-0.747022922201	-0.716468981035	-0.707100957759
7.2	-0.811410765983	-0.766409016264	-0.751677207826	-0.747970431028	-0.716241756510	-0.708971834284
7.5	-0.811243935603	-0.766550386911	-0.750652073548	-0.747487745516	-0.715396856866	-0.710321126603
7.8	-0.810791594374	-0.766071582440	-0.749915955127	-0.745737771758	-0.714200788942	-0.711161288375
8.1	-0.810195604706	-0.765090183933	-0.749119608545	-0.743419380793	-0.713153145608	-0.711252830718
8.4	-0.809550979150	-0.763718920735	-0.748134323779	-0.740927761195	-0.712939555713	-0.710136513698
8.7	-0.808916268269	-0.762061986276	-0.746987609030	-0.738437117905	-0.713007145234	-0.708580171549

9.0	-0.808323642638	-0.760212743953	-0.745733133601	-0.736044239134	-0.713030526987	-0.707146744217
9.3	-0.807787727662	-0.758252469884	-0.744420475152	-0.733809247661	-0.712976127118	-0.706126240556
9.6	-0.807312461078	-0.756250033650	-0.743089997447	-0.731768730646	-0.712852408049	-0.705640424997
9.9	-0.806895896027	-0.754262373487	-0.741773202372	-0.729941408149	-0.712673647570	-0.705572607972
10.2	-0.806533276224	-0.752335427524	-0.740494047830	-0.728331464105	-0.712454625411	-0.705732505087
10.5	-0.806218788558	-0.750505153920	-0.739270131600	-0.726931301494	-0.712209817540	-0.705984457491
10.8	-0.805946537066	-0.748798508684	-0.738113599411	-0.725724691208	-0.711952949271	-0.706250697617
11.1	-0.805710999157	-0.747234258754	-0.737031889771	-0.724690274649	-0.711696140764	-0.706489900911
11.4	-0.805507230762	-0.745823684010	-0.736028472691	-0.723805091009	-0.711448995912	-0.706682506420
11.7	-0.805330886992	-0.744571235535	-0.735103683466	-0.723047356339	-0.711218084523	-0.706822222991
12.0	-0.805178214689	-0.743475281939	-0.734255618149	-0.722398107756	-0.711007048320	-0.706910836128
12.3	-0.805045966689	-0.742529062676	-0.733481031414	-0.721841626329	-0.710817192143	-0.706954784543
12.6	-0.804931370798	-0.741721860800	-0.732776042880	-0.721365107190	-0.710648293978	-0.706962807706
12.9	-0.804832032284	-0.741040296189	-0.732136584432	-0.720957963168	-0.710499284771	-0.706944266303
13.2	-0.804745877089	-0.740469638726	-0.731558609672	-0.720611139015	-0.710368686143	-0.706907989720
13.5	-0.804671139144	-0.739994979787	-0.731038124220	-0.720316665811	-0.710254853480	-0.706861599613
13.8	-0.804606275076	-0.739602065486	-0.730571138088	-0.720067399778	-0.710156104462	-0.706811123009
14.1	-0.804549942653	-0.739277860512	-0.730153627837	-0.719856908934	-0.710070750178	-0.706760924812
14.4	-0.804500985956	-0.739010847123	-0.729781550325	-0.719679452388	-0.709997169146	-0.706713866627
14.7	-0.804458405851	-0.738791094144	-0.729450880244	-0.719529950843	-0.709933835457	-0.706671572394
15.0	-0.804421332391	-0.738610178444	-0.729157670523	-0.719403970578	-0.709879330377	-0.706634722460
15.3	-0.804389009855	-0.738461065572	-0.728898121322	-0.719297682084	-0.709832375741	-0.706603379748
15.6	-0.804360790934	-0.738337937673	-0.728668644410	-0.719207828830	-0.709791857731	-0.706577211363
15.9	-0.804336114729	-0.738236015482	-0.728465909166	-0.719131664072	-0.709756804885	-0.706555694665
16.2	-0.804314503198	-0.738151400327	-0.728286859027	-0.719066901985	-0.709726397624	-0.706538223989
16.5	-0.804295537353	-0.738080921614	-0.728128733104	-0.719011654870	-0.709699956983	-0.706524210498
16.8	-0.804278871571	-0.738022011278	-0.727989045176	-0.718964385304	-0.709676927111	-0.706513104808
17.1	-0.804264195518	-0.737972583432	-0.727865584543	-0.718923822294	-0.709656842080	-0.706504411122
17.4	-0.804251250618	-0.737930958017	-0.727756402467	-0.718888935968	-0.709639321956	-0.706497719147
17.7	-0.804239809278	-0.737895757864	-0.727659760763	-0.718858875547	-0.709624043895	-0.706492664298
18.0	-0.804229679255	-0.737865883687	-0.727574146760	-0.718832931795	-0.709610737699	-0.706488957912
18.3	-0.804220691303	-0.737840424293	-0.727498222109	-0.718810511119	-0.709599154665	-0.706486333993
18.6	-0.804212706302	-0.737818648107	-0.727430823489	-0.718791101679	-0.709589078792	-0.706484592198
18.9	-0.804205595877	-0.737799945500	-0.727370916167	-0.718774270397	-0.709580314789	-0.706483551411
19.2	-0.804199252107	-0.737783826270	-0.727317613995	-0.718759637489	-0.709572679800	-0.706483066167
19.5	-0.804193584905	-0.737769873191	-0.727270125362	-0.718746872872	-0.709566012734	-0.706483012769
19.8	-0.804188504333	-0.737757749525	-0.727227756068	-0.718735693344	-0.709560168861	-0.706483297436
20.1	-0.804183953840	-0.737747179098	-0.727189918538	-0.718725859163	-0.709555021696	-0.706483837270
20.4	-0.804179859680	-0.737737924081	-0.727156079581	-0.718717160884	-0.709550460147	-0.706484561747
20.7	-0.804176172736	-0.737729790121	-0.727125771290	-0.718709428485	-0.709546392592	-0.706485430075
21.0	-0.804172848214	-0.737722617424	-0.727098601682	-0.718702517355	-0.709542742387	-0.706486396434

21.3	-0.804169841704	-0.737716269000	-0.727074200898	-0.718696310571	-0.709539445067	-0.706487427389
21.6	-0.804167121901	-0.737710635655	-0.727052262473	-0.718690713391	-0.709536457336	-0.706488500903
21.9	-0.804164654282	-0.737705617340	-0.727032517481	-0.718685649308	-0.709533731966	-0.706489601982
22.2	-0.804162411707	-0.737701138682	-0.727014714715	-0.718681052987	-0.709531237152	-0.706490710260
22.5	-0.804160374947	-0.737697132263	-0.726998653319	-0.718676874647	-0.709528952055	-0.706491815685
22.8	-0.804158518926	-0.737693534896	-0.726984133382	-0.718673066893	-0.709526851502	-0.706492907472
23.1	-0.804156825408	-0.737690302363	-0.726970996141	-0.718669592544	-0.709524916040	-0.706493979942
23.4	-0.804155277499	-0.737687386143	-0.726959093662	-0.718666419177	-0.709523131764	-0.706495022987
23.7	-0.804153863119	-0.737684756544	-0.726948297967	-0.718663516018	-0.709521484049	-0.706496036600
24.0	-0.804152565753	-0.737682374208	-0.726938486924	-0.718660853026	-0.709519957406	-0.706497011326
24.3	-0.804151371444	-0.737680209884	-0.726929563222	-0.718658405706	-0.709518536169	-0.706497936814
24.6	-0.804150274712	-0.737678241517	-0.726921437045	-0.718656148673	-0.709517209763	-0.706498821707
24.9	-0.804149263947	-0.737676448259	-0.726914024000	-0.718654061936	-0.709515967213	-0.706499660623
25.2	-0.804148330091	-0.737674808572	-0.726907255715	-0.718652124898	-0.709514800139	-0.706500451201
25.5	-0.804147465540	-0.737673304791	-0.726901066159	-0.718650318089	-0.709513695945	-0.706501192177
25.8	-0.804146667122	-0.737671922523	-0.726895402789	-0.718648631229	-0.709512650099	-0.706501878104
26.1	-0.804145925541	-0.737670648796	-0.726890210328	-0.718647047138	-0.709511655622	-0.706502524971
26.4	-0.804145236938	-0.737669473683	-0.726885445561	-0.718645555940	-0.709510703954	-0.706503119202
26.7	-0.804144596105	-0.737668388307	-0.726881066352	-0.718644150980	-0.709509796891	-0.706503676996
27.0	-0.804143996750	-0.737667379232	-0.726877036001	-0.718642822510	-0.709508925959	-0.706504190872
27.3	-0.804143441171	-0.737666443814	-0.726873324574	-0.718641566097	-0.709508093857	-0.706504666189
27.6	-0.804142922952	-0.737665575203	-0.726869907230	-0.718640376061	-0.709507296178	-0.706505106859
27.9	-0.804142436425	-0.737664766480	-0.726866743906	-0.718639252893	-0.709506535061	-0.706505517832
28.2	-0.804141983398	-0.737664015232	-0.726863825745	-0.718638189324	-0.709505807058	-0.706505896845
28.5	-0.804141559781	-0.737663312171	-0.726861125126	-0.718637187240	-0.709505113845	-0.706506250350
28.8	-0.804141164755	-0.737662660083	-0.726858622515	-0.718636244114	-0.709504459035	-0.706506578852
29.1	-0.804140794074	-0.737662051445	-0.726856299198	-0.718635357648	-0.709503837286	-0.706506887341
29.4	-0.804140448401	-0.737661483674	-0.726854149542	-0.718634526222	-0.709503250792	-0.706507174696
29.7	-0.804140125550	-0.737660952016	-0.726852152222	-0.718633748504	-0.709502698354	-0.706507440203
30.0	-0.804139821122	-0.737660458370	-0.726850292483	-0.718633022012	-0.709502178643	-0.706507692284
30.3	-0.804139537471	-0.737659997091	-0.726848564191	-0.718632346635	-0.709501696010	-0.706507929038
30.6	-0.804139270350	-0.737659565286	-0.726846953749	-0.718631718295	-0.709501243065	-0.706508150038
30.9	-0.804139021642	-0.737659164223	-0.726845453855	-0.718631137309	-0.709500823549	-0.706508364877
31.2	-0.804138793462	-0.737658787717	-0.726844060442	-0.718630600929	-0.709500436129	-0.706508559560
31.5	-0.804138570559	-0.737658435706	-0.726842750724	-0.718630103744	-0.709500076483	-0.706508747911
31.8	-0.804138366344	-0.737658104644	-0.726841532541	-0.718629645131	-0.709499743661	-0.706508921192
32.1	-0.804138176826	-0.737657797535	-0.726840396225	-0.718629224985	-0.709499439279	-0.706509087375
32.4	-0.804137998976	-0.737657510230	-0.726839333818	-0.718628836358	-0.709499156289	-0.706509247175
32.7	-0.804137826570	-0.737657239181	-0.726838334215	-0.718628477788	-0.709498897780	-0.706509397766
33.0	-0.804137667321	-0.737656982084	-0.726837401083	-0.718628148101	-0.709498656597	-0.706509536400
33.3	-0.804137516514	-0.737656742070	-0.726836525453	-0.718627841783	-0.709498435797	-0.706509669466

33.6	-0.804137375304	-0.737656518492	-0.726835705725	-0.718627561025	-0.709498233158	-0.706509795391
33.9	-0.804137243843	-0.737656308128	-0.726834937805	-0.718627300282	-0.709498043599	-0.706509913975
34.2	-0.804137116489	-0.737656106142	-0.726834213989	-0.718627057796	-0.709497868754	-0.706510025170
34.5	-0.804136997352	-0.737655918350	-0.726833534873	-0.718626832216	-0.709497707090	-0.706510128359
34.8	-0.804136885668	-0.737655739882	-0.726832897453	-0.718626620600	-0.709497552603	-0.706510228723
35.1	-0.804136779462	-0.737655570824	-0.726832297595	-0.718626424299	-0.709497412025	-0.706510319993
35.4	-0.804136675344	-0.737655410473	-0.726831729442	-0.718626238126	-0.709497278442	-0.706510408615
35.7	-0.804136581785	-0.737655258669	-0.726831199726	-0.718626065019	-0.709497153600	-0.706510487141
36.0	-0.804136491738	-0.737655116215	-0.726830699536	-0.718625901051	-0.709497035532	-0.706510567781
36.3	-0.804136402417	-0.737654976874	-0.726830223953	-0.718625743927	-0.709496921016	-0.706510636834
36.6	-0.804136322064	-0.737654847593	-0.726829780180	-0.718625595331	-0.709496814205	-0.706510703031
36.9	-0.804136243406	-0.737654722409	-0.726829359265	-0.718625454019	-0.709496709907	-0.706510763015
37.2	-0.804136168562	-0.737654604386	-0.726828961737	-0.718625318437	-0.709496612430	-0.706510824197
37.5	-0.804136099917	-0.737654494633	-0.726828588969	-0.718625190288	-0.709496518736	-0.706510879462
37.8	-0.804136028300	-0.737654383017	-0.726828230643	-0.718625067015	-0.709496428662	-0.706510928475
38.1	-0.804135968043	-0.737654283442	-0.726827899870	-0.718624949688	-0.709496341513	-0.706510974678
38.4	-0.804135908116	-0.737654184450	-0.726827584517	-0.718624835865	-0.709496258129	-0.706511017773
38.7	-0.804135849004	-0.737654093175	-0.726827284340	-0.718624726309	-0.709496177358	-0.706511060690
39.0	-0.804135793610	-0.737654001872	-0.726827001072	-0.718624622932	-0.709496098927	-0.706511098810
39.3	-0.804135744352	-0.737653919990	-0.726826736518	-0.718624522896	-0.709496024545	-0.706511133665
39.6	-0.804135696080	-0.737653836919	-0.726826484725	-0.718624425884	-0.709495950601	-0.706511164396
39.9	-0.804135648521	-0.737653760962	-0.726826244514	-0.718624335890	-0.709495883301	-0.706511199188

Table S6: Potential energy curves of $^2\Sigma^+$ states calculated using the (ECP+CPP)FCI method. Distances in atomic units, energies in Hartree.

Table S7: Potential energy curves of $^2\Pi$ states calculated using the (ECP+CPP)FCI method. Distances in atomic units, energies in Hartree.

R	$1^2\Pi$	$2^2\Pi$	$3^2\Pi$	$4^2\Pi$	$5^2\Pi$
	Na(3s)+Sr(5s5p ^{3}P)	Na(3p)+Sr(5s^2 ^{1}S)	Na(3s)+Sr(5s4d ^{3}D)	Na(3s)+Sr(5s4d ^{1}D)	Na(3s)+Sr(5s5p ^{1}P)
3.0	-0.623031048929	-0.599301778060	-0.556407564049	-0.541855963966	-0.525033174048
3.3	-0.653430897157	-0.624405484673	-0.586727832897	-0.570300325041	-0.557593929733
3.6	-0.680336365275	-0.648386418279	-0.613259071165	-0.595797850516	-0.586267281140
3.9	-0.703941106533	-0.671202981195	-0.636194503925	-0.618970993851	-0.609822137202
4.2	-0.724344985795	-0.692377394560	-0.656029956153	-0.639513929437	-0.628843718642
4.5	-0.741422293400	-0.711052112823	-0.673077123768	-0.656537192030	-0.647687195863
4.8	-0.755053418587	-0.726514593920	-0.687398165015	-0.669939061913	-0.666753645810
5.1	-0.765314825851	-0.738517048476	-0.699003898331	-0.682115555324	-0.679882982317

5.4	-0.772501139545	-0.747237848108	-0.708042725112	-0.693541910080	-0.687381213808
5.7	-0.777045035034	-0.753100556457	-0.714816634150	-0.701839205788	-0.693055859097
6.0	-0.779423359254	-0.756614246514	-0.719705724792	-0.707581864933	-0.697944798547
6.3	-0.780091538757	-0.758277255314	-0.723095010964	-0.711345761125	-0.701987192727
6.6	-0.779450953612	-0.758532778827	-0.725331950814	-0.713625911988	-0.705198328871
6.9	-0.777839116903	-0.757755307516	-0.726709939316	-0.714824663266	-0.707609062686
7.2	-0.775531990178	-0.756252266556	-0.727466020954	-0.715260088329	-0.709333712326
7.5	-0.772751578088	-0.754271911833	-0.727784497077	-0.715180727451	-0.710523275202
7.8	-0.769675049704	-0.752012831668	-0.727802420010	-0.714779467000	-0.711311757665
8.1	-0.766443397641	-0.749632564440	-0.727615855874	-0.714204086025	-0.711803131301
8.4	-0.763168908119	-0.747254360645	-0.727286990897	-0.713566362018	-0.712070258727
8.7	-0.759941467946	-0.744971719048	-0.726852607088	-0.712960676427	-0.712146477296
9.0	-0.756833800096	-0.742850693579	-0.726333583363	-0.712527559253	-0.711972780936
9.3	-0.753905659228	-0.740930508059	-0.725744523004	-0.712319346707	-0.711543308911
9.6	-0.751206684580	-0.739223844280	-0.725101617082	-0.712150215941	-0.711066176528
9.9	-0.748777399937	-0.737719353119	-0.724426698549	-0.711962470493	-0.710607209835
10.2	-0.746647976243	-0.736388629985	-0.723746497187	-0.711757263420	-0.710167729318
10.5	-0.744834659723	-0.735197270959	-0.723088118035	-0.711544334705	-0.709740914005
10.8	-0.743335678472	-0.734116586133	-0.722474027418	-0.711333138184	-0.709324179272
11.1	-0.742129764699	-0.733130713341	-0.721918979532	-0.711130695255	-0.708921750077
11.4	-0.741180078337	-0.732236210217	-0.721429709722	-0.710941425200	-0.708542834828
11.7	-0.740442236543	-0.731436036506	-0.721006474798	-0.710767713479	-0.708197699138
12.0	-0.739872445093	-0.730732955856	-0.720645288984	-0.710610595778	-0.707894050524
12.3	-0.739432504386	-0.730125837852	-0.720339843722	-0.710470156585	-0.707635120452
12.6	-0.739091574593	-0.729609129067	-0.720083027336	-0.710345885421	-0.707419859824
12.9	-0.738825820193	-0.729174114508	-0.719867781306	-0.710236824712	-0.707244227877
13.2	-0.738617197728	-0.728810534532	-0.719687568526	-0.710141715111	-0.707102704882
13.5	-0.738452184995	-0.728507935755	-0.719536641178	-0.710059170200	-0.706989510235
13.8	-0.738320671455	-0.728256524470	-0.719410050762	-0.709987770884	-0.706899310263
14.1	-0.738215031173	-0.728047596701	-0.719303603228	-0.709926112764	-0.706827467100
14.4	-0.738129530061	-0.727873682432	-0.719213823117	-0.709872909428	-0.706770188757
14.7	-0.738059803063	-0.727728517099	-0.719137832262	-0.709826988733	-0.706724401808
15.0	-0.738002517336	-0.727606927133	-0.719073266866	-0.709787315727	-0.706687678726

15.3	-0.737955104643	-0.727504675897	-0.719018187354	-0.709752982135	-0.706658106256
15.6	-0.737915589701	-0.727418309797	-0.718971011152	-0.709723220596	-0.706634192763
15.9	-0.737882426584	-0.727345040440	-0.718930447986	-0.709697368253	-0.706614763253
16.2	-0.737854415517	-0.727282594054	-0.718895439223	-0.709674864519	-0.706598904667
16.5	-0.737830606593	-0.727229130670	-0.718865117002	-0.709655240183	-0.706585897169
16.8	-0.737810255117	-0.727183158096	-0.718838770313	-0.709638098370	-0.706575175282
17.1	-0.737792761497	-0.727143455235	-0.718815805025	-0.709623097994	-0.706566296338
17.4	-0.737777648971	-0.727109014708	-0.718795729641	-0.709609950063	-0.706558909736
17.7	-0.737764530402	-0.727079029384	-0.718778130493	-0.709598403086	-0.706552732311
18.0	-0.737753092263	-0.727052815096	-0.718762664566	-0.709588252635	-0.706547546696
18.3	-0.737743076893	-0.727029818169	-0.718749036774	-0.709579305804	-0.706543170890
18.6	-0.737734274143	-0.727009582238	-0.718736999679	-0.709571407941	-0.706539463323
18.9	-0.737726508182	-0.726991701895	-0.718726345140	-0.709564423354	-0.706536311347
19.2	-0.737719633681	-0.726975861955	-0.718716889558	-0.709558233108	-0.706533622569
19.5	-0.737713532371	-0.726961789178	-0.718708480120	-0.709552733170	-0.706531316164
19.8	-0.737708095731	-0.726949242273	-0.718700983683	-0.709547834915	-0.706529332248
20.1	-0.737703242342	-0.726938042001	-0.718694287382	-0.709543460267	-0.706527613060
20.4	-0.737698894099	-0.726928012213	-0.718688287627	-0.709539542435	-0.706526120390
20.7	-0.737694991307	-0.726919003058	-0.718682901462	-0.709536023799	-0.706524821207
21.0	-0.737691482986	-0.726910901193	-0.718678056266	-0.709532858546	-0.706523678688
21.3	-0.737688315011	-0.726903598062	-0.718673684643	-0.709529999668	-0.706522671263
21.6	-0.737685458768	-0.726897001151	-0.718669736045	-0.709527412407	-0.706521780279
21.9	-0.737682869000	-0.726891030365	-0.718666154375	-0.709525059957	-0.706520986814
22.2	-0.737680519086	-0.726885616104	-0.718662897972	-0.709522915299	-0.706520273759
22.5	-0.737678388225	-0.726880701831	-0.718659934208	-0.709520959464	-0.706519637409
22.8	-0.737676444956	-0.726876233351	-0.718657230526	-0.709519167569	-0.706519061918
23.1	-0.737674675055	-0.726872162309	-0.718654754746	-0.709517518820	-0.706518538797
23.4	-0.737673056214	-0.726868447711	-0.718652487345	-0.709516003604	-0.706518067742
23.7	-0.737671577478	-0.726865057532	-0.718650403703	-0.709514601737	-0.706517636896
24.0	-0.737670222074	-0.726861954763	-0.718648485308	-0.709513304209	-0.706517242926
24.3	-0.737668976733	-0.726859109179	-0.718646715234	-0.709512100099	-0.706516887845
24.6	-0.737667833633	-0.726856500468	-0.718645080631	-0.709510979287	-0.706516559214
24.9	-0.737666779443	-0.726854103227	-0.718643570237	-0.709509939023	-0.706516256510

25.2	-0.737665807742	-0.726851896931	-0.718642173503	-0.709508968037	-0.706515980039
25.5	-0.737664912172	-0.726849863208	-0.718640881075	-0.709508065445	-0.706515725341
25.8	-0.737664082293	-0.726847989487	-0.718639683129	-0.709507221081	-0.706515487078
26.1	-0.737663318039	-0.726846257709	-0.718638573799	-0.709506438159	-0.706515270757
26.4	-0.737662608868	-0.726844656291	-0.718637543277	-0.709505703995	-0.706515068248
26.7	-0.737661949141	-0.726843173478	-0.718636589431	-0.709505022052	-0.706514878247
27.0	-0.737661339104	-0.726841796143	-0.718635702791	-0.709504383267	-0.706514703291
27.3	-0.737660773825	-0.726840523929	-0.718634884284	-0.709503792436	-0.706514542619
27.6	-0.737660247951	-0.726839337626	-0.718634121667	-0.709503239928	-0.706514387681
27.9	-0.737659759644	-0.726838234170	-0.718633417273	-0.709502727385	-0.706514248277
28.2	-0.737659303688	-0.726837209191	-0.718632760204	-0.709502248891	-0.706514113400
28.5	-0.737658879962	-0.726836254588	-0.718632151187	-0.709501803997	-0.706513985493
28.8	-0.737658487400	-0.726835366523	-0.718631585228	-0.709501387808	-0.706513871898
29.1	-0.737658118664	-0.726834537218	-0.718631060344	-0.709501003368	-0.706513761437
29.4	-0.737657777021	-0.726833764762	-0.718630571259	-0.709500644066	-0.706513659497
29.7	-0.737657456529	-0.726833044531	-0.718630115419	-0.709500310394	-0.706513560977
30.0	-0.737657158897	-0.726832369603	-0.718629691119	-0.709499997883	-0.706513473040
30.3	-0.737656879281	-0.726831740295	-0.718629297440	-0.709499706810	-0.706513386405
30.6	-0.737656619479	-0.726831150336	-0.718628924627	-0.709499432689	-0.706513310758
30.9	-0.737656375273	-0.726830600209	-0.718628580373	-0.709499177798	-0.706513234676
31.2	-0.737656148778	-0.726830090141	-0.718628258988	-0.709498938089	-0.706513165362
31.5	-0.737655931683	-0.726829603543	-0.718627954476	-0.709498712242	-0.706513099495
31.8	-0.737655733153	-0.726829152427	-0.718627667202	-0.709498496602	-0.706513040306
32.1	-0.737655546866	-0.726828731648	-0.718627403641	-0.709498299265	-0.706512985027
32.4	-0.737655369395	-0.726828337013	-0.718627149505	-0.709498108453	-0.706512928638
32.7	-0.737655202852	-0.726827961351	-0.718626910927	-0.709497927877	-0.706512880615
33.0	-0.737655048129	-0.726827611349	-0.718626686718	-0.709497759087	-0.706512833529
33.3	-0.737654902082	-0.726827281419	-0.718626473364	-0.709497597815	-0.706512790391
33.6	-0.737654763413	-0.726826972104	-0.718626271536	-0.709497442314	-0.706512749562
33.9	-0.737654635040	-0.726826682545	-0.718626083705	-0.709497296806	-0.706512712370
34.2	-0.737654511536	-0.726826406589	-0.718625901597	-0.709497156343	-0.706512678828
34.5	-0.737654396742	-0.726826147467	-0.718625731774	-0.709497025109	-0.706512645841
34.8	-0.737654286296	-0.726825904509	-0.718625566559	-0.709496894860	-0.706512610280

35.1	-0.737654184533	-0.726825674219	-0.718625414084	-0.709496776585	-0.706512580306
35.4	-0.737654086136	-0.726825453593	-0.718625265519	-0.709496658135	-0.706512554245
35.7	-0.737653993081	-0.726825250324	-0.718625129622	-0.709496550309	-0.706512524476
36.0	-0.737653909016	-0.726825056929	-0.718624995749	-0.709496444575	-0.706512501180
36.3	-0.737653823538	-0.726824869944	-0.718624871683	-0.709496344274	-0.706512474470
36.6	-0.737653746735	-0.726824697558	-0.718624752268	-0.709496250784	-0.706512455299
36.9	-0.737653673293	-0.726824532063	-0.718624638007	-0.709496156857	-0.706512434200
37.2	-0.737653603115	-0.726824375230	-0.718624531730	-0.709496072891	-0.706512414474
37.5	-0.737653536762	-0.726824229201	-0.718624430960	-0.709495991570	-0.706512392910
37.8	-0.737653473277	-0.726824084490	-0.718624332078	-0.709495910837	-0.706512380014
38.1	-0.737653415932	-0.726823955177	-0.718624242691	-0.709495841261	-0.706512361694
38.4	-0.737653361504	-0.726823830006	-0.718624156401	-0.709495769882	-0.706512347012
38.7	-0.737653306041	-0.726823709037	-0.718624072568	-0.709495703287	-0.706512331692
39.0	-0.737653255408	-0.726823595235	-0.718623998145	-0.709495644430	-0.706512317147
39.3	-0.737653207599	-0.726823490622	-0.718623923878	-0.709495583368	-0.706512302524
39.6	-0.737653164662	-0.726823390041	-0.718623854319	-0.709495527380	-0.706512290461
39.9	-0.737653120042	-0.726823292911	-0.718623789812	-0.709495476711	-0.706512277115

Table S7: Potential energy curves of $^2\Pi$ states calculated using the (ECP+CPP)FCI method. Distances in atomic units, energies in Hartree.

Table S8: Potential energy curves of $^2\Delta$ states calculated using the (ECP+CPP)FCI method. Distances in atomic units, energies in Hartree.

R	$1^2\Delta$	$2^2\Delta$
	Na(3s)+Sr(5s4d ^{3}D)	Na(3s)+Sr(5s4d ^{1}D)
3.0	-0.603595747470	-0.559908466662
3.3	-0.627711291102	-0.588849663929
3.6	-0.650647062901	-0.613503333764
3.9	-0.671839235818	-0.634202366411
4.2	-0.690874865252	-0.651540197855
4.5	-0.707320990501	-0.665932005765

4.8	-0.720835208288	-0.677547229795
5.1	-0.731311817565	-0.686474349358
5.4	-0.738909118100	-0.692880349757
5.7	-0.743970942923	-0.697044221973
6.0	-0.746923103200	-0.701732114354
6.3	-0.748195484725	-0.705821280739
6.6	-0.748179492034	-0.708766629256
6.9	-0.747211503866	-0.710824323443
7.2	-0.745571025758	-0.712203872078
7.5	-0.743485685285	-0.713073947655
7.8	-0.741138564514	-0.713568366325
8.1	-0.738675404357	-0.713791195364
8.4	-0.736210623805	-0.713821346761
8.7	-0.733832143508	-0.713717176634
9.0	-0.731604983495	-0.713521015187
9.3	-0.729574024862	-0.713263495574
9.6	-0.727766059732	-0.712967269849
9.9	-0.726191691510	-0.712649881324
10.2	-0.724847648964	-0.712325649501
10.5	-0.723719686757	-0.712006470135
10.8	-0.722786180377	-0.711701966838
11.1	-0.722021717414	-0.711419240909
11.4	-0.721400263966	-0.711162761885
11.7	-0.720897306643	-0.710934515262
12.0	-0.720491138382	-0.710734492215
12.3	-0.720163246813	-0.710561215278
12.6	-0.719898301395	-0.710412363363

12.9	-0.719683823675	-0.710285221121
13.2	-0.719509744629	-0.710176989851
13.5	-0.719368032789	-0.710085020702
13.8	-0.719252266568	-0.710006908160
14.1	-0.719157321894	-0.709940509962
14.4	-0.719079134104	-0.709883994766
14.7	-0.719014462963	-0.709835793668
15.0	-0.718960721167	-0.709794581835
15.3	-0.718915838330	-0.709759236776
15.6	-0.718878166254	-0.709728826715
15.9	-0.718846385900	-0.709702580212
16.2	-0.718819435045	-0.709679841143
16.5	-0.718796461887	-0.709660074808
16.8	-0.718776792940	-0.709642843595
17.1	-0.718759860869	-0.709627764174
17.4	-0.718745225491	-0.709614535937
17.7	-0.718732512844	-0.709602888640
18.0	-0.718721427100	-0.709592611342
18.3	-0.718711719441	-0.709583516584
18.6	-0.718703185599	-0.709575441583
18.9	-0.718695655941	-0.709568260464
19.2	-0.718688991019	-0.709561854004
19.5	-0.718683069439	-0.709556124021
19.8	-0.718677789926	-0.709550986226
20.1	-0.718673072954	-0.709546366050
20.4	-0.718668843934	-0.709542205097
20.7	-0.718665044990	-0.709538447566

21.0	-0.718661623520	-0.709535048474
21.3	-0.718658535218	-0.709531966826
21.6	-0.718655740434	-0.709529168721
21.9	-0.718653206086	-0.709526619363
22.2	-0.718650905336	-0.709524299510
22.5	-0.718648814915	-0.709522184385
22.8	-0.718646911667	-0.709520253424
23.1	-0.718645169983	-0.709518481889
23.4	-0.718643582441	-0.709516864466
23.7	-0.718642131238	-0.709515383682
24.0	-0.718640797617	-0.709514018165
24.3	-0.718639572657	-0.709512761454
24.6	-0.718638445419	-0.709511601192
24.9	-0.718637405009	-0.709510527598
25.2	-0.718636446121	-0.709509533590
25.5	-0.718635556375	-0.709508609553
25.8	-0.718634736836	-0.709507751812
26.1	-0.718633973311	-0.709506953847
26.4	-0.718633262755	-0.709506204478
26.7	-0.718632605701	-0.709505508955
27.0	-0.718631988632	-0.709504854149
27.3	-0.718631420931	-0.709504247104
27.6	-0.718630887385	-0.709503676669
27.9	-0.718630387458	-0.709503135964
28.2	-0.718629925175	-0.709502637446
28.5	-0.718629489626	-0.709502165381
28.8	-0.718629085228	-0.709501725030

29.1	-0.718628706092	-0.709501311662
29.4	-0.718628354491	-0.709500928295
29.7	-0.718628021797	-0.709500565789
30.0	-0.718627714621	-0.709500228228
30.3	-0.718627426115	-0.709499912669
30.6	-0.718627154123	-0.709499615788
30.9	-0.718626902473	-0.709499336521
31.2	-0.718626667490	-0.709499082172
31.5	-0.718626446765	-0.709498838391
31.8	-0.718626237963	-0.709498610376
32.1	-0.718626045512	-0.709498399141
32.4	-0.718625863456	-0.709498198875
32.7	-0.718625691220	-0.709498009053
33.0	-0.718625527233	-0.709497828240
33.3	-0.718625376613	-0.709497662402
33.6	-0.718625234521	-0.709497505656
33.9	-0.718625098435	-0.709497358191
34.2	-0.718624972626	-0.709497217881
34.5	-0.718624851318	-0.709497084548
34.8	-0.718624736619	-0.709496955245
35.1	-0.718624630204	-0.709496838338
35.4	-0.718624527464	-0.709496722344
35.7	-0.718624429121	-0.709496614852
36.0	-0.718624338440	-0.709496513172
36.3	-0.718624251295	-0.709496414544
36.6	-0.718624169283	-0.709496320430
36.9	-0.718624089372	-0.709496233408

37.2	-0.718624014668	-0.709496148026
37.5	-0.718623947078	-0.709496069429
37.8	-0.718623876095	-0.709495991295
38.1	-0.718623815809	-0.709495922267
38.4	-0.718623754204	-0.709495854238
38.7	-0.718623696678	-0.709495787434
39.0	-0.718623643587	-0.709495728566
39.3	-0.718623593753	-0.709495670284
39.6	-0.718623542210	-0.709495611147
39.9	-0.718623498025	-0.709495561455

Table S8: Potential energy curves of $^2\Delta$ states calculated using the (ECP+CPP)FCI method. Distances in atomic units, energies in Hartree.

Table S9: Potential energy curves of $^4\Sigma^+$ states calculated using the (ECP+CPP)FCI method. Distances in atomic units, energies in Hartree.

R	$1^4\Sigma^+$<br>Na(3s)+Sr(5s5p ^{3}P)	$2^4\Sigma^+$<br>Na(3s)+Sr(5s4d ^{3}D)
3.0	-0.551052976601	-0.523453106833
3.3	-0.580233970671	-0.555219046299
3.6	-0.605975209545	-0.584180707550
3.9	-0.628627947120	-0.609805144565
4.2	-0.648633064321	-0.631819486297
4.5	-0.666157404716	-0.650044041431
4.8	-0.681133842421	-0.664599473521
5.1	-0.693500171009	-0.676034806311
5.4	-0.703347662658	-0.686007067000
5.7	-0.710926125835	-0.696782934962

6.0	-0.716581868007	-0.706404792156
6.3	-0.720688210132	-0.714044918162
6.6	-0.723641244720	-0.719853921392
6.9	-0.726106789821	-0.723768323933
7.2	-0.728865950140	-0.725534603379
7.5	-0.731339227656	-0.726172596504
7.8	-0.733280843525	-0.726286290102
8.1	-0.734762622674	-0.726088296358
8.4	-0.735882824948	-0.725700451949
8.7	-0.736724955881	-0.725205196268
9.0	-0.737354158339	-0.724659477248
9.3	-0.737819877751	-0.724101804527
9.6	-0.738159424426	-0.723557198625
9.9	-0.738401103453	-0.723041012638
10.2	-0.738566637561	-0.722561880611
10.5	-0.738672892249	-0.722123823743
10.8	-0.738733123422	-0.721727826074
11.1	-0.738757845966	-0.721372868346
11.4	-0.738755457139	-0.721056685939
11.7	-0.738732719915	-0.720776293522
12.0	-0.738695071431	-0.720528381384
12.3	-0.738646903562	-0.720309546843
12.6	-0.738591763582	-0.720116518647
12.9	-0.738532478731	-0.719946277329
13.2	-0.738471284183	-0.719796094066
13.5	-0.738409932716	-0.719663571413
13.8	-0.738349758025	-0.719546623884

14.1	-0.738291727463	-0.719443423753
14.4	-0.738236516501	-0.719352371100
14.7	-0.738184568572	-0.719272036510
15.0	-0.738136117584	-0.719201147434
15.3	-0.738091253166	-0.719138546490
15.6	-0.738049955542	-0.719083216516
15.9	-0.738012116231	-0.719034248529
16.2	-0.737977586634	-0.718990857336
16.5	-0.737946180452	-0.718952364756
16.8	-0.737917699762	-0.718918206735
17.1	-0.737891935211	-0.718887887664
17.4	-0.737868683104	-0.718860992993
17.7	-0.737847727758	-0.718837162481
18.0	-0.737828883779	-0.718816067911
18.3	-0.737811953132	-0.718797417516
18.6	-0.737796764128	-0.718780939527
18.9	-0.737783142769	-0.718766385737
19.2	-0.737770939267	-0.718753520270
19.5	-0.737759998752	-0.718742127552
19.8	-0.737750188904	-0.718732012642
20.1	-0.737741396802	-0.718723004813
20.4	-0.737733506995	-0.718714947984
20.7	-0.737726417368	-0.718707710737
21.0	-0.737720043951	-0.718701186835
21.3	-0.737714306319	-0.718695279068
21.6	-0.737709140887	-0.718689914996
21.9	-0.737704478806	-0.718685031407

22.2	-0.737700271586	-0.718680573123
22.5	-0.737696472374	-0.718676504616
22.8	-0.737693032982	-0.718672781645
23.1	-0.737689920317	-0.718669372736
23.4	-0.737687094906	-0.718666250521
23.7	-0.737684535565	-0.718663386946
24.0	-0.737682205825	-0.718660752750
24.3	-0.737680081946	-0.718658329103
24.6	-0.737678143874	-0.718656090827
24.9	-0.737676373725	-0.718654018164
25.2	-0.737674751535	-0.718652092066
25.5	-0.737673261437	-0.718650293613
25.8	-0.737671889524	-0.718648613132
26.1	-0.737670623106	-0.718647034021
26.4	-0.737669454947	-0.718645546656
26.7	-0.737668374054	-0.718644143648
27.0	-0.737667368025	-0.718642817786
27.3	-0.737666434980	-0.718641562937
27.6	-0.737665568679	-0.718640374016
27.9	-0.737664761202	-0.718639251827
28.2	-0.737664011649	-0.718638188724
28.5	-0.737663308744	-0.718637186830
28.8	-0.737662657981	-0.718636244483
29.1	-0.737662049683	-0.718635358188
29.4	-0.737661482243	-0.718634526961
29.7	-0.737660951162	-0.718633749064
30.0	-0.737660457561	-0.718633022798

30.3	-0.737659997128	-0.718632347119
30.6	-0.737659565643	-0.718631718871
30.9	-0.737659163943	-0.718631138051
31.2	-0.737658787964	-0.718630601209
31.5	-0.737658435662	-0.718630103620
31.8	-0.737658104771	-0.718629644875
32.1	-0.737657797656	-0.718629224718
32.4	-0.737657510485	-0.718628836330
32.7	-0.737657239592	-0.718628477360
33.0	-0.737656982043	-0.718628147818
33.3	-0.737656741835	-0.718627841368
33.6	-0.737656518266	-0.718627560610
33.9	-0.737656308229	-0.718627300372
34.2	-0.737656105691	-0.718627057530
34.5	-0.737655918876	-0.718626831931
34.8	-0.737655739898	-0.718626620239
35.1	-0.737655571324	-0.718626424044
35.4	-0.737655410679	-0.718626237994
35.7	-0.737655259185	-0.718626064944
36.0	-0.737655116125	-0.718625900871
36.3	-0.737654977007	-0.718625743954
36.6	-0.737654848064	-0.718625595395
36.9	-0.737654722995	-0.718625454231
37.2	-0.737654604434	-0.718625318618
37.5	-0.737654495110	-0.718625190426
37.8	-0.737654382550	-0.718625066719
38.1	-0.737654283585	-0.718624949878

38.4	-0.737654184255	-0.718624835893
38.7	-0.737654093680	-0.718624726174
39.0	-0.737654001399	-0.718624623116
39.3	-0.737653920112	-0.718624522925
39.6	-0.737653836823	-0.718624425643
39.9	-0.737653760618	-0.718624335880

Table S9: Potential energy curves of $^4\Sigma^+$ states calculated using the (ECP+CPP)FCI method. Distances in atomic units, energies in Hartree.

Table S10: Potential energy curves of $^4\Pi$ states calculated using the (ECP+CPP)FCI method. Distances in atomic units, energies in Hartree.

R	$1^4\Pi$ Na(3s)+Sr(5s5p ^{3}P)	$2^4\Pi$ Na(3s)+Sr(5s4d ^{3}D)
3.0	-0.576162845329	-0.557518626139
3.3	-0.607359243736	-0.584187571622
3.6	-0.635061079406	-0.607715408622
3.9	-0.659361787752	-0.628013678111
4.2	-0.680506078370	-0.645265618601
4.5	-0.698581635498	-0.661276559443
4.8	-0.713573885005	-0.676079532762
5.1	-0.725532385484	-0.688635369077
5.4	-0.734661387731	-0.699201401953
5.7	-0.741298571460	-0.707599149031
6.0	-0.745846317598	-0.714026847262
6.3	-0.748710098287	-0.718790301787
6.6	-0.750262148898	-0.722193610128
6.9	-0.750826716473	-0.724508333671

7.2	-0.750677454721	-0.725965797651
7.5	-0.750040110060	-0.726758107550
7.8	-0.749096908982	-0.727042947559
8.1	-0.747990948354	-0.726949668863
8.4	-0.746829985360	-0.726585142931
8.7	-0.745690238630	-0.726038415743
9.0	-0.744620773978	-0.725383574836
9.3	-0.743648800408	-0.724680928757
9.6	-0.742785372996	-0.723977167545
9.9	-0.742030741355	-0.723305576631
10.2	-0.741378742174	-0.722687150531
10.5	-0.740819930999	-0.722132563329
10.8	-0.740343672600	-0.721644755682
11.1	-0.739939331701	-0.721221508626
11.4	-0.739596947443	-0.720857632291
11.7	-0.739307517550	-0.720546563727
12.0	-0.739063115624	-0.720281478903
12.3	-0.738856830490	-0.720055823727
12.6	-0.738682731546	-0.719863714939
12.9	-0.738535765857	-0.719699999681
13.2	-0.738411619708	-0.719560233479
13.5	-0.738306649267	-0.719440678570
13.8	-0.738217795334	-0.719338184091
14.1	-0.738142450847	-0.719250088235
14.4	-0.738078444951	-0.719174181643
14.7	-0.738023951986	-0.719108611827
15.0	-0.737977446037	-0.719051828235

15.3	-0.737937643874	-0.719002529203
15.6	-0.737903488747	-0.718959624226
15.9	-0.737874087982	-0.718922203511
16.2	-0.737848708254	-0.718889496163
16.5	-0.737826731027	-0.718860851733
16.8	-0.737807649318	-0.718835722626
17.1	-0.737791030119	-0.718813637850
17.4	-0.737776514728	-0.718794195923
17.7	-0.737763801058	-0.718777050889
18.0	-0.737752635016	-0.718761908436
18.3	-0.737742799777	-0.718748510592
18.6	-0.737734115366	-0.718736635753
18.9	-0.737726424784	-0.718726095518
19.2	-0.737719596333	-0.718716719930
19.5	-0.737713523749	-0.718708365781
19.8	-0.737708102353	-0.718700906838
20.1	-0.737703258056	-0.718694236839
20.4	-0.737698913330	-0.718688255176
20.7	-0.737695010150	-0.718682881041
21.0	-0.737691501672	-0.718678043950
21.3	-0.737688331490	-0.718673677195
21.6	-0.737685473600	-0.718669732089
21.9	-0.737682881605	-0.718666152805
22.2	-0.737680529160	-0.718662897612
22.5	-0.737678396950	-0.718659934183
22.8	-0.737676451431	-0.718657231241
23.1	-0.737674680917	-0.718654755680

23.4	-0.737673060124	-0.718652488044
23.7	-0.737671580552	-0.718650404805
24.0	-0.737670225192	-0.718648486491
24.3	-0.737668978828	-0.718646716153
24.6	-0.737667835466	-0.718645081681
24.9	-0.737666781405	-0.718643570662
25.2	-0.737665808928	-0.718642174145
25.5	-0.737664913222	-0.718640881758
25.8	-0.737664082975	-0.718639683906
26.1	-0.737663318854	-0.718638574398
26.4	-0.737662609768	-0.718637543708
26.7	-0.737661949441	-0.718636589959
27.0	-0.737661339392	-0.718635702931
27.3	-0.737660773631	-0.718634884868
27.6	-0.737660248682	-0.718634122057
27.9	-0.737659759681	-0.718633417837
28.2	-0.737659303515	-0.718632760571
28.5	-0.737658880683	-0.718632151531
28.8	-0.737658487997	-0.718631585525
29.1	-0.737658118316	-0.718631060729
29.4	-0.737657777239	-0.718630571593
29.7	-0.737657457140	-0.718630115398
30.0	-0.737657158935	-0.718629691068
30.3	-0.737656879230	-0.718629297489
30.6	-0.737656619112	-0.718628924541
30.9	-0.737656375093	-0.718628580449
31.2	-0.737656149328	-0.718628259298

31.5	-0.737655931254	-0.718627954610
31.8	-0.737655732984	-0.718627667261
32.1	-0.737655546649	-0.718627404006
32.4	-0.737655369733	-0.718627149699
32.7	-0.737655202342	-0.718626911152
33.0	-0.737655047947	-0.718626686879
33.3	-0.737654902457	-0.718626473199
33.6	-0.737654763270	-0.718626271573
33.9	-0.737654635029	-0.718626083881
34.2	-0.737654511078	-0.718625901519
34.5	-0.737654396338	-0.718625731935
34.8	-0.737654286464	-0.718625566580
35.1	-0.737654184986	-0.718625413902
35.4	-0.737654085687	-0.718625265359
35.7	-0.737653993257	-0.718625129930
36.0	-0.737653909678	-0.718624995611
36.3	-0.737653823867	-0.718624871939
36.6	-0.737653746759	-0.718624752236
36.9	-0.737653673726	-0.718624638280
37.2	-0.737653603251	-0.718624531787
37.5	-0.737653537144	-0.718624431039
37.8	-0.737653472806	-0.718624332344
38.1	-0.737653415718	-0.718624242690
38.4	-0.737653361829	-0.718624156553
38.7	-0.737653305858	-0.718624072569
39.0	-0.737653254890	-0.718623998088
39.3	-0.737653207235	-0.718623924053

39.6	-0.737653165056	-0.718623854602
39.9	-0.737653119705	-0.718623789896

Table S10: Potential energy curve of $^4\Pi$ state calculated using the (ECP+CPP)FCI method. Distances in atomic units, energies in Hartree.

Table S11: Potential energy curve of $1^4\Delta$ state calculated using the (ECP+CPP)FCI method. Distances in atomic units, energies in Hartree.

R	$1^4\Delta$
	Na(3s)+Sr(5s4d ^{3}D)
3.0	-0.572492274676
3.3	-0.606988712971
3.6	-0.635747265745
3.9	-0.659164020855
4.2	-0.677860357273
4.5	-0.692367766836
4.8	-0.703147765423
5.1	-0.710664399723
5.4	-0.715409618862
5.7	-0.717883489195
6.0	-0.718560343742
6.3	-0.717862336042
6.6	-0.716146787171
6.9	-0.716103251626
7.2	-0.717744939191
7.5	-0.718885749585
7.8	-0.719646077469
8.1	-0.720124545180

8.4	-0.720398872443
8.7	-0.720528577500
9.0	-0.720558183317
9.3	-0.720520441424
9.6	-0.720439120222
9.9	-0.720331284121
10.2	-0.720209066427
10.5	-0.720080946380
10.8	-0.719952753720
11.1	-0.719828360403
11.4	-0.719710251788
11.7	-0.719599884372
12.0	-0.719498026380
12.3	-0.719404911016
12.6	-0.719320448615
12.9	-0.719244321996
13.2	-0.719176045203
13.5	-0.719115078407
13.8	-0.719060820082
14.1	-0.719012648161
14.4	-0.718969967908
14.7	-0.718932208785
15.0	-0.718898832251
15.3	-0.718869336901
15.6	-0.718843273014
15.9	-0.718820237561
16.2	-0.718799864639

16.5	-0.718781832127
16.8	-0.718765869585
17.1	-0.718751714282
17.4	-0.718739156209
17.7	-0.718727996088
18.0	-0.718718068796
18.3	-0.718709224733
18.6	-0.718701334259
18.9	-0.718694283091
19.2	-0.718687973814
19.5	-0.718682316468
19.8	-0.718677232038
20.1	-0.718672660901
20.4	-0.718668539366
20.7	-0.718664819862
21.0	-0.718661457089
21.3	-0.718658412947
21.6	-0.718655649502
21.9	-0.718653139502
22.2	-0.718650855889
22.5	-0.718648778737
22.8	-0.718646884893
23.1	-0.718645150368
23.4	-0.718643568105
23.7	-0.718642120411
24.0	-0.718640789555
24.3	-0.718639566752

24.6	-0.718638441163
24.9	-0.718637401573
25.2	-0.718636443759
25.5	-0.718635554623
25.8	-0.718634735806
26.1	-0.718633972509
26.4	-0.718633262459
26.7	-0.718632605139
27.0	-0.718631988105
27.3	-0.718631420574
27.6	-0.718630887061
27.9	-0.718630387577
28.2	-0.718629924933
28.5	-0.718629489582
28.8	-0.718629085477
29.1	-0.718628706111
29.4	-0.718628354751
29.7	-0.718628021929
30.0	-0.718627714581
30.3	-0.718627426109
30.6	-0.718627154115
30.9	-0.718626902819
31.2	-0.718626667496
31.5	-0.718626446744
31.8	-0.718626237892
32.1	-0.718626045347
32.4	-0.718625863307

32.7	-0.718625691408
33.0	-0.718625527625
33.3	-0.718625376543
33.6	-0.718625234733
33.9	-0.718625098249
34.2	-0.718624972765
34.5	-0.718624851464
34.8	-0.718624736603
35.1	-0.718624630023
35.4	-0.718624527836
35.7	-0.718624428911
36.0	-0.718624338585
36.3	-0.718624251186
36.6	-0.718624169644
36.9	-0.718624089530
37.2	-0.718624014797
37.5	-0.718623947213
37.8	-0.718623876199
38.1	-0.718623815683
38.4	-0.718623754119
38.7	-0.718623696718
39.0	-0.718623643455
39.3	-0.718623593621
39.6	-0.718623542304
39.9	-0.718623498055

Table S11: Potential energy curve of $1^4\Delta$ state calculated using the (ECP+CPP)FCI method. Distances in atomic units, energies in Hartree.

Table S12: Transition dipole moments (TDM) in $a_0 e_0$ units for transitions between the ground state $X(1)^2\Sigma^+$ and subsequent excited $^2\Sigma^+$ states calculated using the (ECP+CPP)FCI method. Distances are given in atomic units.

R	X–$2^2\Sigma^+$	X–$3^2\Sigma^+$	X–$4^2\Sigma^+$	X–$5^2\Sigma^+$	X–$6^2\Sigma^+$
3.0	0.589529E+00	-0.231041E+00	-0.724542E-01	-0.362060E+00	-0.623979E+00
3.3	-0.662665E+00	-0.218605E+00	-0.175181E+00	-0.357698E+00	-0.432981E+00
3.6	-0.737622E+00	-0.212891E+00	-0.194708E+00	-0.394315E+00	-0.169652E-01
3.9	-0.828924E+00	-0.221538E+00	-0.167953E+00	-0.452025E+00	0.508751E+00
4.2	-0.930527E+00	-0.248873E+00	-0.170830E+00	-0.575726E+00	0.776819E+00
4.5	-0.102436E+01	-0.294609E+00	-0.205968E+00	-0.110744E+01	0.206626E+00
4.8	-0.109603E+01	-0.350048E+00	-0.188432E+00	-0.120699E+01	0.728779E+00
5.1	-0.114117E+01	-0.403359E+00	0.113725E+01	-0.757032E+00	0.116288E+01
5.4	-0.116206E+01	-0.445647E+00	0.132837E+01	-0.888295E+00	0.113392E+01
5.7	-0.116335E+01	-0.471952E+00	0.146971E+01	-0.140932E+01	0.725908E+00
6.0	-0.114985E+01	-0.480553E+00	0.161683E+01	-0.180576E+01	0.113743E+00
6.3	-0.112573E+01	-0.472050E+00	0.176704E+01	-0.189656E+01	0.229544E+00
6.6	-0.109443E+01	-0.448415E+00	0.191263E+01	-0.186136E+01	0.392205E+00
6.9	-0.105888E+01	-0.412350E+00	0.204630E+01	-0.181582E+01	0.376029E+00
7.2	-0.102165E+01	-0.366959E+00	0.216271E+01	-0.178576E+01	0.204966E+00
7.5	-0.985204E+00	-0.315508E+00	0.225800E+01	-0.177462E+01	0.178994E+00
7.8	-0.951966E+00	-0.261184E+00	0.232745E+01	-0.178785E+01	0.751181E+00
8.1	-0.924397E+00	-0.206837E+00	0.235919E+01	-0.184151E+01	0.113524E+01
8.4	-0.904946E+00	-0.154803E+00	0.231229E+01	-0.198289E+01	0.131336E+01
8.7	-0.895998E+00	-0.106800E+00	0.200662E+01	-0.235678E+01	0.140289E+01
9.0	-0.899785E+00	-0.639045E-01	0.881522E+00	-0.301494E+01	0.144654E+01
9.3	-0.918251E+00	-0.265697E-01	0.104708E+00	-0.318234E+01	0.145762E+01
9.6	-0.952831E+00	-0.527536E-02	0.401041E+00	-0.319923E+01	0.144112E+01

9.9	-0.100409E+01	0.319936E-01	0.457858E+00	-0.322968E+01	0.139940E+01
10.2	-0.107124E+01	0.539179E-01	0.424282E+00	-0.326974E+01	0.133390E+01
10.5	-0.115180E+01	0.711147E-01	0.353745E+00	-0.331060E+01	0.124602E+01
10.8	-0.124159E+01	0.834101E-01	0.273673E+00	-0.334673E+01	0.113810E+01
11.1	-0.133566E+01	0.906498E-01	0.199855E+00	-0.337514E+01	0.101430E+01
11.4	-0.142938E+01	0.929873E-01	0.139867E+00	-0.339443E+01	0.880923E+00
11.7	-0.151933E+01	0.910119E-01	0.953671E-01	-0.340425E+01	0.745709E+00
12.0	-0.160335E+01	0.856683E-01	0.645890E-01	-0.340520E+01	0.616322E+00
12.3	-0.168038E+01	0.780573E-01	0.444090E-01	-0.339875E+01	0.498742E+00
12.6	-0.175007E+01	0.692303E-01	0.316747E-01	-0.338684E+01	0.396383E+00
12.9	-0.181257E+01	0.600532E-01	0.237774E-01	-0.337145E+01	0.310197E+00
13.2	-0.186833E+01	0.511531E-01	0.188095E-01	-0.335427E+01	0.239357E+00
13.5	-0.191796E+01	0.429275E-01	0.154878E-01	-0.333651E+01	0.182077E+00
13.8	-0.196213E+01	0.355870E-01	0.130268E-01	-0.331900E+01	0.136232E+00
14.1	-0.200149E+01	0.292060E-01	0.109670E-01	-0.330221E+01	0.997519E-01
14.4	-0.203667E+01	0.237695E-01	0.908405E-02	-0.328639E+01	0.708042E-01
14.7	-0.206820E+01	0.192099E-01	0.727233E-02	-0.327163E+01	0.478527E-01
15.0	-0.209658E+01	0.154332E-01	0.550277E-02	-0.325795E+01	0.296544E-01
15.3	-0.212221E+01	0.123362E-01	0.378268E-02	-0.324528E+01	0.152243E-01
15.6	-0.214546E+01	0.981754E-02	0.213015E-02	-0.323358E+01	0.379230E-02
15.9	-0.216662E+01	0.778305E-02	0.565803E-03	-0.322275E+01	0.523522E-02
16.2	-0.218595E+01	0.614923E-02	0.885724E-03	-0.321273E+01	0.123111E-01
16.5	-0.220367E+01	0.484354E-02	0.221851E-02	-0.320344E+01	0.177758E-01
16.8	-0.221997E+01	0.380451E-02	0.341876E-02	-0.319480E+01	0.218796E-01
17.1	-0.223499E+01	0.298087E-02	0.448026E-02	-0.318676E+01	0.247996E-01
17.4	-0.224889E+01	0.233010E-02	0.539978E-02	-0.317927E+01	0.266608E-01
17.7	-0.226176E+01	0.181751E-02	0.617938E-02	-0.317227E+01	0.275487E-01

18.0	-0.227373E+01	0.141483E-02	0.682030E-02	-0.316572E+01	0.275314E-01
18.3	-0.228486E+01	0.109936E-02	0.732903E-02	-0.315958E+01	0.266786E-01
18.6	-0.229525E+01	0.852763E-03	0.771266E-02	-0.315382E+01	0.250830E-01
18.9	-0.230495E+01	0.660384E-03	0.798117E-02	-0.314841E+01	0.228735E-01
19.2	-0.231403E+01	0.510666E-03	0.814295E-02	-0.314332E+01	0.202161E-01
19.5	-0.232254E+01	0.394335E-03	0.821160E-02	-0.313852E+01	0.172991E-01
19.8	-0.233052E+01	0.304152E-03	0.819598E-02	-0.313400E+01	0.143081E-01
20.1	-0.233802E+01	0.234306E-03	0.810987E-02	-0.312972E+01	0.114030E-01
20.4	-0.234507E+01	0.180274E-03	0.796395E-02	-0.312569E+01	0.870059E-02
20.7	-0.235171E+01	0.138549E-03	0.776935E-02	-0.312187E+01	0.627215E-02
21.0	-0.235797E+01	0.106423E-03	0.753418E-02	-0.311826E+01	0.414970E-02
21.3	-0.236388E+01	0.816789E-04	0.727117E-02	-0.311484E+01	0.233519E-02
21.6	-0.236945E+01	0.626147E-04	0.698514E-02	-0.311160E+01	0.811205E-03
21.9	-0.237473E+01	0.479693E-04	0.668363E-02	-0.310852E+01	0.449715E-03
22.2	-0.237971E+01	0.367483E-04	0.637335E-02	-0.310560E+01	0.148008E-02
22.5	-0.238444E+01	0.281163E-04	0.605790E-02	-0.310283E+01	0.231204E-02
22.8	-0.238891E+01	0.214820E-04	0.574173E-02	-0.310019E+01	0.297624E-02
23.1	-0.239316E+01	0.164453E-04	0.542675E-02	-0.309769E+01	0.350024E-02
23.4	-0.239718E+01	0.125500E-04	0.511669E-02	-0.309530E+01	0.390829E-02
23.7	-0.240101E+01	0.956780E-05	0.481039E-02	-0.309303E+01	0.422038E-02
24.0	-0.240464E+01	0.729170E-05	0.451237E-02	-0.309087E+01	0.445406E-02
24.3	-0.240810E+01	0.556600E-05	0.421968E-02	-0.308880E+01	0.462400E-02
24.6	-0.241139E+01	0.422620E-05	0.393510E-02	-0.308684E+01	0.474201E-02
24.9	-0.241452E+01	0.322000E-05	0.365924E-02	-0.308496E+01	0.481773E-02
25.2	-0.241750E+01	0.247570E-05	0.339103E-02	-0.308317E+01	0.485897E-02
25.5	-0.242035E+01	0.188030E-05	0.313140E-02	-0.308146E+01	0.487230E-02
25.8	-0.242306E+01	0.142690E-05	0.288007E-02	-0.307982E+01	0.486254E-02

26.1	-0.242565E+01	0.108460E-05	0.263686E-02	-0.307826E+01	0.483502E-02
26.4	-0.242812E+01	0.816500E-06	0.240224E-02	-0.307676E+01	0.479257E-02
26.7	-0.243048E+01	0.623700E-06	0.217727E-02	-0.307533E+01	0.473852E-02
27.0	-0.243274E+01	0.478200E-06	0.195988E-02	-0.307396E+01	0.467465E-02
27.3	-0.243490E+01	0.362200E-06	0.175329E-02	-0.307265E+01	0.460304E-02
27.6	-0.243696E+01	0.284100E-06	0.155417E-02	-0.307139E+01	0.452530E-02
27.9	-0.243894E+01	0.215800E-06	0.136431E-02	-0.307019E+01	0.444278E-02
28.2	-0.244083E+01	0.164200E-06	0.118391E-02	-0.306903E+01	0.435644E-02
28.5	-0.244265E+01	0.124600E-06	0.101231E-02	-0.306793E+01	0.426716E-02
28.8	-0.244439E+01	0.999000E-07	0.849259E-03	-0.306686E+01	0.417587E-02
29.1	-0.244606E+01	0.758000E-07	0.695102E-03	-0.306584E+01	0.408357E-02
29.4	-0.244766E+01	0.600000E-07	0.549172E-03	-0.306486E+01	0.398981E-02
29.7	-0.244919E+01	0.477000E-07	0.411484E-03	-0.306392E+01	0.389467E-02
30.0	-0.245067E+01	0.372000E-07	0.281162E-03	-0.306301E+01	0.380058E-02
30.3	-0.245208E+01	0.279000E-07	0.159104E-03	-0.306214E+01	0.370550E-02
30.6	-0.245345E+01	0.223000E-07	0.423178E-04	-0.306130E+01	0.361116E-02
30.9	-0.245476E+01	0.231000E-07	0.658135E-04	-0.306049E+01	0.351761E-02
31.2	-0.245601E+01	0.188000E-07	0.168601E-03	-0.305972E+01	0.342458E-02
31.5	-0.245723E+01	0.150000E-07	0.264744E-03	-0.305897E+01	0.333248E-02
31.8	-0.245839E+01	0.530000E-08	0.356259E-03	-0.305825E+01	0.324143E-02
32.1	-0.245952E+01	0.160000E-08	0.441199E-03	-0.305756E+01	0.315151E-02
32.4	-0.246060E+01	0.100000E-09	0.521888E-03	-0.305689E+01	0.306298E-02
32.7	-0.246164E+01	-0.120000E-08	0.596875E-03	-0.305624E+01	0.297562E-02
33.0	-0.246264E+01	-0.160000E-08	0.668353E-03	-0.305562E+01	0.288977E-02
33.3	-0.246361E+01	-0.120000E-08	0.735871E-03	-0.305502E+01	0.280548E-02
33.6	-0.246455E+01	-0.260000E-08	0.799597E-03	-0.305444E+01	0.272269E-02
33.9	-0.246545E+01	-0.300000E-09	0.859260E-03	-0.305388E+01	0.264157E-02

34.2	-0.246632E+01	-0.500000E-09	0.914995E-03	-0.305334E+01	0.256211E-02
34.5	-0.246716E+01	0.303000E-06	0.967104E-03	-0.305283E+01	0.251081E-02
34.8	-0.246797E+01	0.370000E-08	0.101788E-02	-0.305232E+01	0.240847E-02
35.1	-0.246875E+01	-0.702000E-07	0.106361E-02	-0.305184E+01	0.236337E-02
35.4	-0.246951E+01	-0.112000E-07	0.110810E-02	-0.305136E+01	0.228079E-02
35.7	-0.247024E+01	0.964000E-07	0.114886E-02	-0.305091E+01	0.221235E-02
36.0	-0.247095E+01	0.370000E-08	0.118864E-02	-0.305047E+01	0.212311E-02
36.3	-0.247163E+01	0.160000E-08	0.122387E-02	-0.305004E+01	0.205657E-02
36.6	-0.247230E+01	0.600000E-09	0.125758E-02	-0.304963E+01	0.199184E-02
36.9	-0.247294E+01	0.600000E-09	0.128929E-02	-0.304923E+01	0.192905E-02
37.2	-0.247356E+01	-0.210000E-07	0.131871E-02	-0.304884E+01	0.186818E-02
37.5	-0.247416E+01	-0.161000E-07	0.134498E-02	-0.304847E+01	0.180921E-02
37.8	-0.247474E+01	-0.124000E-07	0.137085E-02	-0.304811E+01	0.175209E-02
38.1	-0.247530E+01	-0.980000E-08	0.139356E-02	-0.304776E+01	0.169687E-02
38.4	-0.247585E+01	-0.830000E-08	0.141496E-02	-0.304742E+01	0.164347E-02
38.7	-0.247638E+01	-0.750000E-08	0.143452E-02	-0.304709E+01	0.159192E-02
39.0	-0.247689E+01	-0.730000E-08	0.145151E-02	-0.304677E+01	0.154215E-02
39.3	-0.247739E+01	-0.580000E-08	0.146771E-02	-0.304646E+01	0.149411E-02
39.6	-0.247787E+01	-0.460000E-08	0.148283E-02	-0.304615E+01	0.144786E-02
39.9	-0.247834E+01	-0.370000E-08	0.149511E-02	-0.304586E+01	0.140319E-02

Table S12: Transition dipole moments (TDM) in a_0e_0 units for transitions between the ground state X(1)$^2\Sigma^+$ and subsequent excited $^2\Sigma^+$ states calculated using the (ECP+CPP)FCI method. Distances are given in atomic units.

Table S13: Transition dipole moments (TDM) in a_0e_0 units for transitions between the ground state X(1)$^2\Sigma^+$ and subsequent excited $^2\Pi$ states calculated using the (ECP+CPP)FCI method. Distances are given in atomic units.

R	X–1$^2\Pi$	X–2$^2\Pi$	X–3$^2\Pi$	X–4$^2\Pi$	X–5$^2\Pi$

3.0	-0.430507E+00	0.177269E+01	-0.822009E+00	-0.191815E+01	0.275018E+00
3.3	-0.360791E+00	-0.173110E+01	0.343373E+00	0.219130E+01	-0.185909E+00
3.6	-0.285836E+00	-0.161651E+01	0.190304E+00	0.237092E+01	-0.226633E+00
3.9	-0.231360E+00	-0.146557E+01	0.771140E+00	0.248982E+01	-0.118176E+00
4.2	-0.216566E+00	-0.131800E+01	0.120443E+01	0.254714E+01	0.601985E+00
4.5	-0.237244E+00	-0.120419E+01	0.148018E+01	0.263293E+01	0.568678E+00
4.8	-0.272715E+00	-0.113458E+01	0.163076E+01	0.275683E+01	0.521342E+00
5.1	-0.305349E+00	-0.110869E+01	0.167719E+01	0.290831E+01	0.475870E+00
5.4	-0.326873E+00	-0.112817E+01	0.163108E+01	0.307893E+01	0.432640E+00
5.7	-0.335436E+00	-0.119719E+01	0.149411E+01	0.325905E+01	0.396313E+00
6.0	-0.332250E+00	-0.129513E+01	0.127820E+01	0.343683E+01	0.369905E+00
6.3	-0.319737E+00	-0.134477E+01	0.107761E+01	0.359749E+01	0.354633E+00
6.6	-0.300647E+00	-0.130207E+01	0.105258E+01	0.371893E+01	0.350753E+00
6.9	-0.277625E+00	-0.120449E+01	0.132546E+01	0.374899E+01	0.358998E+00
7.2	-0.252956E+00	-0.109213E+01	0.208459E+01	0.349709E+01	0.383771E+00
7.5	-0.228421E+00	-0.984999E+00	0.325714E+01	0.255829E+01	0.442658E+00
7.8	-0.205216E+00	-0.891208E+00	0.389180E+01	0.154152E+01	0.605216E+00
8.1	-0.183976E+00	-0.813108E+00	0.409057E+01	0.975852E+00	0.105687E+01
8.4	-0.164878E+00	-0.750135E+00	0.415195E+01	0.653092E+00	0.134397E+01
8.7	-0.147817E+00	-0.700271E+00	0.415925E+01	0.443621E+00	0.136256E+01
9.0	-0.132551E+00	-0.660861E+00	0.413834E+01	0.296318E+00	0.132863E+01
9.3	-0.118822E+00	-0.629093E+00	0.410063E+01	0.189396E+00	0.127495E+01
9.6	-0.106404E+00	-0.602237E+00	0.405256E+01	0.112313E+00	0.120905E+01
9.9	-0.951242E-01	-0.577770E+00	0.399825E+01	0.591654E-01	0.113470E+01
10.2	-0.848542E-01	-0.553434E+00	0.394050E+01	0.259417E-01	0.105482E+01
10.5	-0.755024E-01	-0.527304E+00	0.388118E+01	0.920542E-02	0.971924E+00

10.8	-0.669961E-01	-0.497904E+00	0.382158E+01	0.547338E-02	0.888313E+00
11.1	-0.592790E-01	-0.464342E+00	0.376254E+01	0.110405E-01	0.806083E+00
11.4	-0.523009E-01	-0.426446E+00	0.370464E+01	0.221505E-01	0.727227E+00
11.7	-0.460160E-01	-0.384819E+00	0.364822E+01	0.353535E-01	0.653645E+00
12.0	-0.403796E-01	-0.340749E+00	0.359352E+01	0.478764E-01	0.587020E+00
12.3	-0.353481E-01	-0.295974E+00	0.354066E+01	0.578533E-01	0.528545E+00
12.6	-0.308764E-01	-0.252334E+00	0.348977E+01	0.643523E-01	0.478685E+00
12.9	-0.269212E-01	-0.211443E+00	0.344092E+01	0.672306E-01	0.437099E+00
13.2	-0.234374E-01	-0.174472E+00	0.339421E+01	0.668916E-01	0.402796E+00
13.5	-0.203825E-01	-0.142065E+00	0.334970E+01	0.640280E-01	0.374429E+00
13.8	-0.177139E-01	-0.114391E+00	0.330740E+01	0.594124E-01	0.350587E+00
14.1	-0.153914E-01	-0.912624E-01	0.326732E+01	0.537609E-01	0.330024E+00
14.4	-0.133768E-01	-0.722639E-01	0.322941E+01	0.476596E-01	0.311745E+00
14.7	-0.116338E-01	-0.568724E-01	0.319359E+01	0.415448E-01	0.295029E+00
15.0	-0.101288E-01	-0.445389E-01	0.315978E+01	0.357133E-01	0.279397E+00
15.3	-0.883111E-02	-0.347402E-01	0.312787E+01	0.303454E-01	0.264551E+00
15.6	-0.771233E-02	-0.270084E-01	0.309777E+01	0.255335E-01	0.250330E+00
15.9	-0.674761E-02	-0.209400E-01	0.306937E+01	0.213069E-01	0.236655E+00
16.2	-0.591488E-02	-0.161975E-01	0.304257E+01	0.176541E-01	0.223503E+00
16.5	-0.519472E-02	-0.125043E-01	0.301727E+01	0.145381E-01	0.210883E+00
16.8	-0.457062E-02	-0.963628E-02	0.299338E+01	0.119078E-01	0.198816E+00
17.1	-0.402854E-02	-0.741445E-02	0.297083E+01	0.970771E-02	0.187322E+00
17.4	-0.355625E-02	-0.569677E-02	0.294951E+01	0.788077E-02	0.176419E+00
17.7	-0.314418E-02	-0.437118E-02	0.292937E+01	0.637386E-02	0.166116E+00
18.0	-0.278376E-02	-0.334978E-02	0.291032E+01	0.513751E-02	0.156415E+00
18.3	-0.246782E-02	-0.256398E-02	0.289230E+01	0.412836E-02	0.147303E+00
18.6	-0.219053E-02	-0.196020E-02	0.287525E+01	0.330791E-02	0.138762E+00

18.9	-0.194684E-02	-0.149685E-02	0.285911E+01	0.264377E-02	0.130767E+00
19.2	-0.173218E-02	-0.114175E-02	0.284382E+01	0.210802E-02	0.123288E+00
19.5	-0.154312E-02	-0.869899E-03	0.282933E+01	0.167724E-02	0.116290E+00
19.8	-0.137628E-02	-0.662159E-03	0.281559E+01	0.133188E-02	0.109743E+00
20.1	-0.122890E-02	-0.503430E-03	0.280256E+01	0.105584E-02	0.103611E+00
20.4	-0.109829E-02	-0.382405E-03	0.279020E+01	0.835774E-03	0.978643E-01
20.7	-0.982645E-03	-0.290319E-03	0.277846E+01	0.660476E-03	0.924754E-01
21.0	-0.879821E-03	-0.220119E-03	0.276732E+01	0.521386E-03	0.874169E-01
21.3	-0.788389E-03	-0.166739E-03	0.275673E+01	0.411156E-03	0.826686E-01
21.6	-0.706907E-03	-0.126304E-03	0.274666E+01	0.323931E-03	0.782091E-01
21.9	-0.634171E-03	-0.956202E-04	0.273709E+01	0.254955E-03	0.740226E-01
22.2	-0.569332E-03	-0.724797E-04	0.272798E+01	0.200565E-03	0.700923E-01
22.5	-0.511230E-03	-0.548182E-04	0.271932E+01	0.157930E-03	0.664054E-01
22.8	-0.459336E-03	-0.415047E-04	0.271107E+01	0.123989E-03	0.629468E-01
23.1	-0.413018E-03	-0.314989E-04	0.270321E+01	0.972946E-04	0.597048E-01
23.4	-0.371584E-03	-0.238516E-04	0.269572E+01	0.764014E-04	0.566684E-01
23.7	-0.334658E-03	-0.181087E-04	0.268858E+01	0.599771E-04	0.538248E-01
24.0	-0.301742E-03	-0.137834E-04	0.268177E+01	0.470659E-04	0.511626E-01
24.3	-0.272419E-03	-0.105150E-04	0.267527E+01	0.367505E-04	0.486693E-01
24.6	-0.246369E-03	-0.808980E-05	0.266907E+01	0.287035E-04	0.463358E-01
24.9	-0.223107E-03	-0.617370E-05	0.266315E+01	0.225963E-04	0.441511E-01
25.2	-0.202522E-03	-0.472390E-05	0.265750E+01	0.175810E-04	0.421040E-01
25.5	-0.184113E-03	-0.365350E-05	0.265209E+01	0.136847E-04	0.401853E-01
25.8	-0.167680E-03	-0.281980E-05	0.264693E+01	0.106607E-04	0.383857E-01
26.1	-0.153013E-03	-0.219170E-05	0.264199E+01	0.826780E-05	0.366980E-01
26.4	-0.139816E-03	-0.169580E-05	0.263726E+01	0.646010E-05	0.351135E-01
26.7	-0.127971E-03	-0.135080E-05	0.263273E+01	0.499670E-05	0.336259E-01

27.0	-0.117261E-03	-0.105190E-05	0.262840E+01	0.384380E-05	0.322272E-01
27.3	-0.107552E-03	-0.811000E-06	0.262424E+01	0.301660E-05	0.309106E-01
27.6	-0.986954E-04	-0.665200E-06	0.262027E+01	0.231270E-05	0.296729E-01
27.9	-0.906394E-04	-0.517100E-06	0.261645E+01	0.179320E-05	0.285069E-01
28.2	-0.832589E-04	-0.412200E-06	0.261279E+01	0.136870E-05	0.274090E-01
28.5	-0.764984E-04	-0.333900E-06	0.260928E+01	0.107550E-05	0.263730E-01
28.8	-0.703095E-04	-0.260100E-06	0.260591E+01	0.806600E-06	0.253951E-01
29.1	-0.645971E-04	-0.208400E-06	0.260268E+01	0.612400E-06	0.244725E-01
29.4	-0.593470E-04	-0.165800E-06	0.259957E+01	0.456500E-06	0.235997E-01
29.7	-0.544852E-04	-0.138900E-06	0.259658E+01	0.368400E-06	0.227727E-01
30.0	-0.500720E-04	-0.118700E-06	0.259371E+01	0.290200E-06	0.219901E-01
30.3	-0.460597E-04	-0.958000E-07	0.259095E+01	0.211500E-06	0.212477E-01
30.6	-0.423481E-04	-0.894000E-07	0.258830E+01	0.159800E-06	0.205423E-01
30.9	-0.390218E-04	-0.715000E-07	0.258574E+01	0.120800E-06	0.198719E-01
31.2	-0.359582E-04	-0.654000E-07	0.258328E+01	0.904000E-07	0.192327E-01
31.5	-0.332967E-04	-0.629000E-07	0.258091E+01	0.699000E-07	0.186238E-01
31.8	-0.308300E-04	-0.448000E-07	0.257863E+01	0.773000E-07	0.180414E-01
32.1	-0.286894E-04	-0.370000E-07	0.257643E+01	0.625000E-07	0.174863E-01
32.4	-0.268261E-04	-0.312000E-07	0.257431E+01	0.470000E-07	0.169548E-01
32.7	-0.252092E-04	-0.262000E-07	0.257227E+01	0.362000E-07	0.164463E-01
33.0	-0.238851E-04	-0.212000E-07	0.257030E+01	0.256000E-07	0.159575E-01
33.3	-0.227747E-04	-0.159000E-07	0.256839E+01	0.201000E-07	0.154889E-01
33.6	-0.219059E-04	-0.143000E-07	0.256656E+01	0.159000E-07	0.150396E-01
33.9	-0.212172E-04	-0.178000E-07	0.256479E+01	0.117000E-07	0.146082E-01
34.2	-0.207575E-04	-0.141000E-07	0.256308E+01	0.880000E-08	0.141931E-01
34.5	-0.204862E-04	-0.106000E-07	0.256142E+01	0.640000E-08	0.137947E-01
34.8	-0.203815E-04	-0.880000E-08	0.255983E+01	0.440000E-08	0.134125E-01

35.1	-0.204575E-04	-0.720000E-08	0.255828E+01	0.220000E-08	0.130443E-01
35.4	-0.206716E-04	-0.640000E-08	0.255679E+01	0.400000E-09	0.126905E-01
35.7	-0.209877E-04	-0.440000E-08	0.255535E+01	0.170000E-08	0.123506E-01
36.0	-0.215011E-04	-0.600000E-08	0.255395E+01	0.280000E-08	0.120240E-01
36.3	-0.220562E-04	-0.490000E-08	0.255260E+01	0.560000E-08	0.117099E-01
36.6	-0.227110E-04	-0.500000E-08	0.255129E+01	0.510000E-08	0.114078E-01
36.9	-0.234655E-04	-0.520000E-08	0.255003E+01	0.540000E-08	0.111173E-01
37.2	-0.242870E-04	-0.590000E-08	0.254880E+01	0.610000E-08	0.108392E-01
37.5	-0.251264E-04	-0.510000E-08	0.254762E+01	0.690000E-08	0.105716E-01
37.8	-0.260245E-04	-0.440000E-08	0.254647E+01	0.240000E-08	0.103137E-01
38.1	-0.269388E-04	-0.470000E-08	0.254535E+01	0.550000E-08	0.100661E-01
38.4	-0.278661E-04	-0.460000E-08	0.254427E+01	0.800000E-08	0.982806E-02
38.7	-0.287906E-04	-0.490000E-08	0.254323E+01	0.970000E-08	0.959946E-02
39.0	-0.297175E-04	-0.500000E-08	0.254221E+01	0.107000E-07	0.937873E-02
39.3	-0.306050E-04	-0.470000E-08	0.254123E+01	0.107000E-07	0.916736E-02
39.6	-0.314782E-04	-0.500000E-08	0.254027E+01	0.108000E-07	0.896268E-02
39.9	-0.323489E-04	-0.450000E-08	0.253935E+01	0.105000E-07	0.876641E-02

Table S13: Transition dipole moments (TDM) in a_0e_0 units for transitions between the ground state X(1)$^2\Sigma^+$ and subsequent excited $^2\Sigma^+$ states calculated using the (ECP+CPP)FCI method. Distances are given in atomic units.

Table S14: The permanent dipole moment (PDM) of NaSr in the X(1)$^2\Sigma^+$ state calculated using the (ECP+CPP)FCI method. Distance in atomic units, PDM in a_0e_0.

R	PDM
3.0	-0.481605E+00
3.3	0.426542E+00
3.6	0.383020E+00
3.9	0.336326E+00

4.2	0.278829E+00
4.5	0.213483E+00
4.8	0.148720E+00
5.1	0.918084E-01
5.4	0.469804E-01
5.7	0.159623E-01
6.0	0.119152E-02
6.3	0.541630E-02
6.6	0.180815E-02
6.9	-0.187171E-01
7.2	-0.434326E-01
7.5	-0.740819E-01
7.8	-0.108886E+00
8.1	-0.146209E+00
8.4	-0.184552E+00
8.7	-0.222459E+00
9.0	-0.258352E+00
9.3	-0.290311E+00
9.6	-0.315905E+00
9.9	-0.332261E+00
10.2	-0.336580E+00
10.5	-0.327203E+00
10.8	-0.304774E+00
11.1	-0.272550E+00
11.4	-0.235298E+00
11.7	-0.197554E+00
12.0	-0.162472E+00

12.3	-0.131665E+00
12.6	-0.105592E+00
12.9	-0.840511E-01
13.2	-0.665367E-01
13.5	-0.524486E-01
13.8	-0.412023E-01
14.1	-0.322746E-01
14.4	-0.252179E-01
14.7	-0.196594E-01
15.0	-0.152941E-01
15.3	-0.118746E-01
15.6	-0.920232E-02
15.9	-0.711851E-02
16.2	-0.549691E-02
16.5	-0.423748E-02
16.8	-0.326119E-02
17.1	-0.250580E-02
17.4	-0.192235E-02
17.7	-0.147251E-02
18.0	-0.112623E-02
18.3	-0.860150E-03
18.6	-0.656028E-03
18.9	-0.499697E-03
19.2	-0.380124E-03
19.5	-0.288819E-03
19.8	-0.219141E-03
20.1	-0.166139E-03

20.4	-0.125798E-03
20.7	-0.951595E-04
21.0	-0.719241E-04
21.3	-0.543328E-04
21.6	-0.409802E-04
21.9	-0.309165E-04
22.2	-0.232662E-04
22.5	-0.175123E-04
22.8	-0.131766E-04
23.1	-0.991180E-05
23.4	-0.747280E-05
23.7	-0.560450E-05
24.0	-0.420140E-05
24.3	-0.316700E-05
24.6	-0.237230E-05
24.9	-0.177820E-05
25.2	-0.131940E-05
25.5	-0.987200E-06
25.8	-0.747300E-06
26.1	-0.566000E-06
26.4	-0.435500E-06
26.7	-0.323300E-06
27.0	-0.232100E-06
27.3	-0.173600E-06
27.6	-0.133300E-06
27.9	-0.100000E-06
28.2	-0.769000E-07

28.5	-0.571000E-07
28.8	-0.480000E-07
29.1	-0.390000E-07
29.4	-0.281000E-07
29.7	-0.243000E-07
30.0	-0.168000E-07
30.3	-0.135000E-07
30.6	-0.860000E-08
30.9	-0.660000E-08
31.2	-0.470000E-08
31.5	-0.370000E-08
31.8	-0.180000E-08
32.1	-0.320000E-08
32.4	-0.170000E-08
32.7	-0.800000E-09
33.0	0.610000E-08
33.3	0.420000E-08
33.6	0.300000E-08
33.9	0.800000E-09
34.2	0.110000E-08
34.5	-0.704000E-07
34.8	-0.110000E-08
35.1	0.970000E-08
35.4	0.344000E-07
35.7	0.840000E-08
36.0	0.210000E-08
36.3	0.190000E-08

36.6	0.130000E-08
36.9	-0.152000E-07
37.2	-0.115000E-07
37.5	-0.800000E-08
37.8	-0.520000E-08
38.1	-0.320000E-08
38.4	-0.190000E-08
38.7	-0.900000E-09
39.0	-0.100000E-09
39.3	0.600000E-09
39.6	0.900000E-09
39.9	0.130000E-08

Table S14: The permanent dipole moment (PDM) of NaSr in the $X(1)^2\Sigma^+$ state calculated using the (ECP+CPP)FCI method. Distance in atomic units, PDM in $a_0 e_0$.

References

(1) Prascher, B. P.; Woon, D. E.; Peterson, K. A.; Dunning, T. H.; Wilson, A. K. Gaussian basis sets for use in correlated molecular calculations. VII. Valence, core-valence, and scalar relativistic basis sets for Li, Be, Na, and Mg. *Theor. Chem. Acc.* **2010**, *128*, 69–82.

(2) Hill, J. G.; Peterson, K. A. Gaussian basis sets for use in correlated molecular calculations. XI. Pseudopotential-based and all-electron relativistic basis sets for alkali metal (K–Fr) and alkaline earth (Ca–Ra) elements. *J. Chem. Phys.* **2017**, *147*, 244106.

(3) Gronowski, M.; Koza, A. M.; Tomza, M. Ab initio properties of the NaLi molecule in the $a^3\Sigma^+$ electronic state. *Phys. Rev. A* **2020**, *102*, 020801.

(4) Shaw, R. A.; Hill, J. G. Midbond basis functions for weakly bound complexes. *Mol. Phys.* **2018**, *116*, 1460–1470.

(5) Tao, F.; Pan, Y. Möller–Plesset perturbation investigation of the He_2 potential and the role of midbond basis functions. *J. Chem. Phys.* **1992**, *97*, 4989–4995.

(6) Knowles, P. J.; Hampel, C.; Werner, H. Coupled cluster theory for high spin, open shell reference wave functions. *J. Chem. Phys.* **1993**, *99*, 5219–5227.

(7) Knowles, P. J.; Hampel, C.; Werner, H.-J. Erratum: "Coupled cluster theory for high spin, open shell reference wave functions" [J. Chem. Phys. 99, 5219 (1993)]. *J. Chem. Phys.* **2000**, *112*, 3106–3107.

(8) Werner, H.-J.; Knowles, P. J.; Manby, F. R.; Black, J. A.; Doll, K.; Heßelmann, A.; Kats, D.; Köhn, A.; Korona, T.; Kreplin, D. A.; et al., The MOLPRO quantum chemistry package. *J. Chem. Phys.* **2020**, *152*, 144107.

(9) Werner, H.-J.; Knowles, P. J.; Celani, P.; Györffy, W.; Hesselmann, A.; Kats, D.; Knizia, G.; Köhn, A.; Korona, T.; Kreplin, D.; et al., MOLPRO, a package of ab initio programs. See url=https://www.molpro.net.

(10) Watts, J. D.; Bartlett, R. J. The coupled-cluster single, double, and triple excitation model for open-shell single reference functions. *J. Chem. Phys.* **1990**, *93*, 6104–6105.

(11) Helgaker, T.; Klopper, W.; Koch, H.; Noga, J. Basis-set convergence of correlated calculations on water. *J. Chem. Phys.* **1997**, *106*, 9639–9646.

(12) Prochnow, E.; Harding, M. E.; Gauss, J. Parallel Calculation of CCSDT and Mk-MRCCSDT Energies. *J. Chem. Theory Comput.* **2010**, *6*, 2339–2347.

(13) Matthews, D. A.; Cheng, L.; Harding, M. E.; Lipparini, F.; Stopkowicz, S.; Jagau, T.-C.; Szalay, P. G.; Gauss, J.; Stanton, J. F. Coupled-cluster techniques for computational chemistry: The CFOUR program package. *J. Chem. Phys.* **2020**, *152*, 214108.

(14) Stanton, J. F.; Gauss, J.; Cheng, L.; Harding, M. E.; Matthews, D. A.; Szalay, P. G. CFOUR, Coupled-Cluster techniques for Computational Chemistry, a quantum-chemical program package. With contributions from A. Asthana, A.A. Auer, R.J. Bartlett, U. Benedikt, C. Berger, D.E. Bernholdt, S. Blaschke, Y. J. Bomble, S. Burger, O. Christiansen, et al., and the integral packages MOLECULE (J. Almlöf and P.R. Taylor), PROPS (P.R. Taylor), ABACUS (T. Helgaker, H.J. Aa. Jensen, P. Jørgensen, and J. Olsen), and ECP routines by A. V. Mitin and C. van Wüllen. For the current version, see http://www.cfour.de.

(15) Kállay, M.; Gauss, J. Approximate treatment of higher excitations in coupled-cluster theory. *J. Chem. Phys.* **2005**, *123*, 214105.

(16) Kállay, M.; Nagy, P. R.; Mester, D.; Rolik, Z.; Samu, G.; Csontos, J.; Csóka, J.; Szabó, P. B.; Gyevi-Nagy, L.; et al., The MRCC program system: Accurate quantum chemistry from water to proteins. *J. Chem. Phys.* **2020**, *152*, 074107.

(17) Kállay, M.; Nagy, P. R.; Mester, D.; Gyevi-Nagy, L.; Csóka, J.; Szabó, P. B.; Rolik, Z.; Samu, G.; Hégely, B.; Ladóczki, B.; et al., MRCC, a quantum chemical program suite. See www.mrcc.hu.

(18) Kramida, A.; Ralchenko, Y. NIST Atomic Spectra Database, NIST Standard Reference Database 78. 1999.

(19) Stanton, J. F.; Bartlett, R. J. The equation of motion coupled-cluster method. A systematic biorthogonal approach to molecular excitation energies, transition probabilities, and excited state properties. *J. Chem. Phys.* **1993**, *98*, 7029–7039.

(20) Kucharski, S. A.; Włoch, M.; Musiał, M.; Bartlett, R. J. Coupled-cluster theory for excited electronic states: The full equation-of-motion coupled-cluster single, double, and triple excitation method. *J. Chem. Phys.* **2001**, *115*, 8263–8266.

(21) Bomble, Y. J.; Sattelmeyer, K. W.; Stanton, J. F.; Gauss, J. On the vertical excitation energy of cyclopentadiene. *J. Chem. Phys.* **2004**, *121*, 5236–5240.

(22) Matthews, D. A.; Stanton, J. F. A new approach to approximate equation-of-motion coupled cluster with triple excitations. *J. Chem. Phys.* **2016**, *145*, 124102.

(23) Stanton, J. F.; Gauss, J. A simple scheme for the direct calculation of ionization potentials with coupled-cluster theory that exploits established excitation energy methods. *J. Chem. Phys.* **1999**, *111*, 8785–8788.

(24) Christiansen, O.; Koch, H.; Jörgensen, P. Response functions in the CC3 iterative triple excitation model. *J. Chem. Phys.* **1995**, *103*, 7429–7441.

(25) Knowles, P.; Handy, N. A new determinant-based full configuration interaction method. *Chem. Phys. Lett.* **1984**, *111*, 315–321.

(26) Knowles, P. J.; Handy, N. C. A determinant based full configuration interaction program. *Comput. Phys. Commun.* **1989**, *54*, 75–83.

(27) Kállay, M.; Gauss, J. Calculation of excited-state properties using general coupled-cluster and configuration-interaction models. *J. Chem. Phys.* **2004**, *121*, 9257–9269.

(28) Cheng, L.; Gauss, J. Analytic energy gradients for the spin-free exact two-component theory using an exact block diagonalization for the one-electron Dirac Hamiltonian. *J. Chem. Phys.* **2011**, *135*, 084114.

(29) Szczepkowski, J.; Gronowski, M.; Grochola, A.; Jastrzebski, W.; Tomza, M.; Kowalczyk, P. Excited Electronic States of Sr_2: Ab Initio Predictions and Experimental Observation of the $2^1\Sigma_u^+$ State. *J. Phys. Chem. A* **2023**, *127*, 4473–4482.

(30) Pototschnig, J. V.; Meyer, R.; Hauser, A. W.; Ernst, W. E. Vibronic transitions in the alkali-metal (Li, Na, K, Rb) – alkaline-earth-metal (Ca, Sr) series: A systematic

analysis of de-excitation mechanisms based on the graphical mapping of Frank-Condon integrals. *Phys. Rev. A* **2017**, *95*, 022501.

(31) Zeid, I.; Atallah, T.; Kontar, S.; Chmaisani, W.; El-Kork, N.; Korek, M. Theoretical electronic structure of the molecules SrX (X= Li, Na, K) toward laser cooling study. *Comput. Theor. Chem.* **2018**, *1126*, 16–32.
